# RiboReport - benchmarking tools for ribosome profiling-based identification of open reading frames in bacteria

**DOI:** 10.1093/bib/bbab549

**Published:** 2022-01-17

**Authors:** Rick Gelhausen, Teresa Müller, Sarah L Svensson, Omer S Alkhnbashi, Cynthia M Sharma, Florian Eggenhofer, Rolf Backofen

**Affiliations:** Bioinformatics Group, Department of Computer Science, University of Freiburg, Georges-Köhler-Allee 106, 79110, Freiburg, Germany; Bioinformatics Group, Department of Computer Science, University of Freiburg, Georges-Köhler-Allee 106, 79110, Freiburg, Germany; Department of Molecular Infection Biology II, Institute of Molecular Infection Biology (IMIB), University of Würzburg, Josef-Schneider-Str. 2 / D15, 97080, Würzburg, Germany; Information and Computer Science Department, King Fahd University of Petroleum and Minerals, Saudi Arabia; SDAIA-KFUPM Joint Research Center for Artificial Intelligence (JRC-AI), King Fahd University of Petroleum and Minerals, Saudi Arabia; Department of Molecular Infection Biology II, Institute of Molecular Infection Biology (IMIB), University of Würzburg, Josef-Schneider-Str. 2 / D15, 97080, Würzburg, Germany; Bioinformatics Group, Department of Computer Science, University of Freiburg, Georges-Köhler-Allee 106, 79110, Freiburg, Germany; Bioinformatics Group, Department of Computer Science, University of Freiburg, Georges-Köhler-Allee 106, 79110, Freiburg, Germany; Signalling Research Centres BIOSS and CIBSS, University of Freiburg, Schänzlestr. 18, 79104, State, Germany

**Keywords:** Ribo-seq, small proteins, ribosome profiling, benchmark, bacteria

## Abstract

Small proteins encoded by short open reading frames (ORFs) with 50 codons or fewer are emerging as an important class of cellular macromolecules in diverse organisms. However, they often evade detection by proteomics or *in silico* methods. Ribosome profiling (Ribo-seq) has revealed widespread translation in genomic regions previously thought to be non-coding, driving the development of ORF detection tools using Ribo-seq data. However, only a handful of tools have been designed for bacteria, and these have not yet been systematically compared. Here, we aimed to identify tools that use Ribo-seq data to correctly determine the translational status of annotated bacterial ORFs and also discover novel translated regions with high sensitivity. To this end, we generated a large set of annotated ORFs from four diverse bacterial organisms, manually labeled for their translation status based on Ribo-seq data, which are available for future benchmarking studies. This set was used to investigate the predictive performance of seven Ribo-seq-based ORF detection tools (REPARATION_blast, DeepRibo, Ribo-TISH, PRICE, smORFer, ribotricer and SPECtre), as well as IRSOM, which uses coding potential and RNA-seq coverage only. DeepRibo and REPARATION_blast robustly predicted translated ORFs, including sORFs, with no significant difference for ORFs in close proximity to other genes versus stand-alone genes. However, no tool predicted a set of novel, experimentally verified sORFs with high sensitivity. Start codon predictions with smORFer show the value of initiation site profiling data to further improve the sensitivity of ORF prediction tools in bacteria. Overall, we find that bacterial tools perform well for sORF detection, although there is potential for improving their performance, applicability, usability and reproducibility.

## 1 Introduction

Identification and characterization of the proteome is crucial for understanding the biology of viruses and cellular organisms, including bacteria. While mass spectrometry (MS) has been the classical genome-wide approach for protein discovery, it often requires pre-existing protein-coding gene or open reading frame (ORF) annotations, can be of limited sensitivity, and is strongly influenced by the biochemistry of each protein species. Small proteins (here defined as those $\leq 50$ amino acids, aa) are especially difficult to detect by MS [1, 2]. The limited sequence information content of their encoding small ORFs (sORFs) makes them challenging to predict using *in silico* approaches, although novel sequence-based tools, as well as improved proteomics analysis methods, are emerging to provide better access to the small proteome [3–8]. In addition, it is becoming apparent that ORFs of ’canonical’ length can even harbour short protein-coding genes hidden in/out-of-frame or even encoded on the opposite strand. These might also be challenging to detect via sequence analysis [9–13]. Small ORFs are therefore likely under-represented in most current bacterial genome annotations [14, 15], despite emerging evidence that they play central roles in diverse physiological processes, including those underlying virulence [2, 15, 16].

Translation is the last step in protein biosynthesis that utilizes RNA, and the power of RNA-seq technology has led to the development of the ribosome profiling (Ribo-seq) approach to detect putative protein-encoding genes based on translation of their mRNAs [17]. Ribo-seq provides a snapshot of the ‘translatome’, which is defined as the set of of actively translated transcripts in the cell. Ribo-seq coverage therefore serves as a proxy for protein expression. This snapshot is generated by high throughput sequencing of so-called ribosome footprints: mRNA fragments that are generated, after halting translation, by nuclease digestion of RNA not protected by the ribosome. In parallel, the total transcriptome is also sequenced to help to define untranslated regions (UTRs) and estimate the available mRNA input for translation. In this way, ORF boundaries can also be defined since Ribo-seq reads are restricted to coding regions. Ribo-seq can also be modified by applying specific inhibitors that target initiating ribosomes at the start codon (e.g. harringtonine/lactimidomycin in eukaryotes [18] or retapamulin/oncocin in bacteria [19, 20]), which restricts ribosome footprints to those of initiating ribosomes. This allows the mapping of translation initiation sites (TISs) and start codons and thereby can reveal ORFs hidden within ORFs and increase confidence in the reading frame. In addition to detecting translation of annotated ORFs, Ribo-seq can also identify novel ORFs missed in genome annotations and proteomic studies. For example, the large number of apparently non-coding transcripts discovered in bacteria by RNA-seq can be reinvestigated for their coding potential [21]. Ribo-seq is especially powerful for detection of sORFs [22], and data from diverse organisms, including bacteria, archaea, yeast, mammalian cells, viruses and even mixed bacterial communities, has identified a wealth of previously unappreciated coding potential, which is often enriched in sORFs [18, 23–27](reviewed in [28]).

Despite its power, challenges arise in the experimental set-up and analysis of Ribo-seq data to generate robust ORF predictions for downstream characterization. Several groups have provided guidelines for application of Ribo-seq to bacterial species [28–30]. Initially, measures such as translation efficiency (TE), also termed ribosome coverage value [31, 32], which is defined as the ratio of ribosome footprint to total transcriptome coverage, were employed to quantitatively detect coding regions. However, this approach can produce high false positive rates [33].

Various groups have developed computational tools that use Ribo-seq coverage patterns and other sequence features for robust identification of translated ORFs (Table 1). These can be grouped into two categories: prediction pipelines and stand-alone prediction tools. ORF prediction pipelines (e.g. Proteoformer [34], HRIBO [35]) incorporate a variety of steps like preprocessing with trimming and mapping, quality control as well as postprocessing (e.g. differential expression analysis). Importantly, such pipelines include (multiple) stand-alone or built-in ORF prediction tools.

**Table 1:** **Overview of identified ORF detection tools**. Most tools make no statement about the taxonomic domain they were developed for. Some, however, utilize eukaryotic data as proof-of-principle (indicated by $^*$). The first eight tools were benchmarked in this manuscript.

Name	Input data	Method	Availability	Taxonomy
DeepRibo [41]	Ribo-seq	Deep Learning	github	Prokaryotes
REPARATION_blast [42]	Ribo-seq	Random Forest	bioconda, github	Prokaryotes
SPECtre [37]	Ribo-seq	Spectral Coherence	github	Eukaryotes$^*$
Ribo-TISH [36]	Ribo-seq	Negative Binominal Test	bioconda, github	Eukaryotes$^*$
IRSOM [21]	RNA-seq	Self-Organizing Map	gitlab, webservice	Eu-, Prokaryotes
smORFer [44]	Ribo-seq	Fourier transform	github	Eu-, Prokaryotes
PRICE [38]	Ribo-seq	EM-algorithm and statistical testing	github	Eukaryotes$^*$
ribotricer [39]	Ribo-seq	3D to 2D projection for periodicity	bioconda, github	Eukaryotes$^*$
RiboTaper [47]	Ribo-/RNA-seq	Multitaper Spectral Analysis	bioconda, galaxy	Eukaryotes$^*$
RiboHMM [48]	Ribo-/RNA-seq	Hidden Markov Models	github	Eukaryotes
ORFrater [49]	Ribo-seq	Linear Regression	github	Eukaryotes$^*$
RibORF [50]	Ribo-seq	Logistic Regression	github	Eukaryotes$^*$
Rp-Bp [51]	Ribo-seq	Markov Chain–Monte Carlo	github	Eukaryotes$^*$

ORF prediction tools vary in their methods, but are commonly designed and tested using eukaryotic Ribo-seq data. Ribo-TISH [36], which was developed for eukaryotes, tests ORFs with a nonparametric Wilcoxon rank-sum test on the read count difference for each nucleotide position to determine the translated ORF. SPECtre [37] is based on spectral coherence to predict regions of active translation from mapped Ribo-seq data. It matches the periodic reading frame function with the signal of aligned reads using a Welch’s spectral density estimate to compute SPECtre scores. Distributions of these scores are then used to assign a posterior probability that predicts if a given region is translated. PRICE [38] (Probabilistic inference of codons activities by an Expectation-Maximization (EM) algorithm) filters noise from the read signal with an EM algorithm. The filtered start codons are then classified by a logistic regression model to identify candidates with active translation and tested for significance using a binomial distribution. ribotricer [39] uses a novel method to detect three-nucleotide periodicity in coverage. For each codon of the profile, the tool searches for a ‘high-low-low’ pattern. The pattern is determined by the transformation of a 3D codon vector to a 2D unit vector, which calculates a phase-score that distinguishes between active and nonactive translation by the help of a cutoff. Predetermined cutoffs are available for different eukaryotes.

Experimental challenges have mostly precluded the use of three-nucleotide periodicity in bacteria [40]. Instead, bacterial tools have so far relied on detection of coverage and sequence features using machine learning [41, 42]. Bacterial genomes also present unique characteristics that can interfere with computational ORF predictions, including high coding density with overlapping genes, unique translation initiation signals and leaderless transcripts. To the best of our knowledge there have been three tools specifically designed for bacteria. REPARATION [42] trains a random forest classifier on all possible ATG-, GTG- and TTG-initiating ORFs. Candidates below a minimum RPKM (reads per kilobase million) cutoff for footprint coverage, determined by the lower bend point of a sigmoid curve, are considered as noise and removed from the prediction. After training, the REPARATION classifier is then used on all potential ORFs satisfying the thresholds. The second tool, DeepRibo [41], uses a convolutional network with a one-hot encoding [43] of the DNA sequence to detect sequence motifs such as the Shine–Dalgarno sequence. This network is then combined with a recurrent neural network architecture to model the patterns in Ribo-seq coverage. DeepRibo models have been trained on Ribo-seq datasets from several bacterial species. DeepRibo also uses the same noise filtering strategy based on a sigmoid curve as REPARATION. Recently, a modular tool for ORF prediction based on both Ribo-seq and TIS data (smORFer [44]) was introduced, which incorporates three-nucleotide periodicity information. The first module generates all potential sORF candidates, which can be filtered by Fourier transformation of their Ribo-seq read signal and/or based on a region of interest. The next two modules are optional and can add confidence to sORF candidate selection. The first uses a read count threshold and tests for three nucleotide periodicity, with an optional filter based on calibrated alignment files. The second module uses TIS data to aid selection of the best start codon for candidates.

Approaches designed to evaluate the coding potential using RNA-seq transcriptome data only, such as CPAT [45], CPC2 [46], and IRSOM [21], have also been developed. Since these cannot use Ribo-seq-specific features like three nucleotide periodicity, they rely on, e.g. sequence or RNA-seq coverage features. IRSOM, established in eukaryotes, uses multiple features such as read distribution over different regions of the ORF, as well as length and reading frame properties. Additionally, sequence features, e.g. nucleotide and k-mer motif frequencies, GC content, and codon properties, are used to create a supervised classifier based on self-organizing maps with a fully connected perceptron layer.

**Figure 1:**
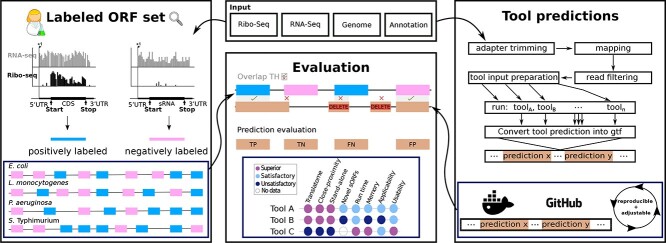
**Overview of the Benchmark approach.** The main contributions of this study are summarized in this figure. First, we provide human labeled benchmark ORF sets for four organisms. To the best of our knowledge, these datasets were not used for the development of ORF prediction tools so far, and therefore provide a valuable resource for the community (left blue box). Second, we provide a complete workflow where future novel tools can be easily tested (blue box, right). Finally, we compare the predictive performance and secondary measures of eight tools with our benchmark set of prokaryotic ORFs and corresponding Ribo-seq data.

As the above tools have not yet been benchmarked together on bacterial data, their broad utility in these organisms is unclear. While DeepRibo and REPARATION have been compared previously, they were compared with the datasets used to train the default model of DeepRibo [41]. In this study, we have identified and compared stand-alone tools for their utility in discovering ORFs from bacterial Ribo-seq datasets, with a special focus on sORFs (Figure 1). Importantly, we used bacterial datasets that were not used for development of any of the tools. Moreover, a large set of verified novel ORFs is necessary to make a statistically meaningful observation. We therefore generated a novel benchmark ORF set manually curated for translation based on Ribo-seq data from four diverse organisms.

We then used these to quantify and compare the performance of ORF prediction tools (seven Ribo-seq-based and one RNA-seq based) that we found could handle bacterial data. All stand-alone tools were integrated into our ORF prediction pipeline (HRIBO [35]) to standardize preprocessing steps. This way, we avoided bias from different adapter trimming or mapping tools. We also tested how well the tools can identify a set of bacterial sORFs that were only recently identified and validated [20]. Finally, we compared tool applicability, usability, and reproducibility to provide a complete picture of their utility. Our benchmark shows clear superiority of tools designed for bacteria, and we make recommendations for tool selection and future developments.

## 2 Materials and Methods

### Ribosome profiling of *E. coli*

#### Growth of bacteria

The *E. coli* MG1655 wild-type strain was grown and harvested for Ribo-seq essentially as described previously [25]. Cultures were grown to mid-log phase (OD_600_ approx. 0.4) in 200 ml lysogeny broth (LB) at $37^\circ \textrm{C}$ with shaking at 200 rpm. A sample for total RNA was transferred to RNA stop mix [95% ethanol, 5% buffer-saturated phenol (Roth)] and snap-frozen in liquid N_2_. Bacteria were then treated with 100 $\mu $g/ml chloramphenicol (final concentration, Sigma) for 2 min at $37^\circ \textrm{C}$, followed by harvest via rapid filtration through a 0.45 $\mu $m PES (polyethersulfone) membrane (Millipore) and immediate freezing in liquid N_2_.

#### Cell harvest

Harvested cells were processed for Ribo-seq as described previously [25] with minor modifications. Frozen cells were resuspended in chilled lysis buffer (100 mM NH_4_Cl, 10 mM MgCl_2_, 20 mM Tris-HCl, pH 8, 0.1% NP-40, 0.4% Triton X-100, 1 mM chloramphenicol) supplemented with 50 U DNase I (Thermo Fisher Scientific) and 500 U RNase inhibitor (moloX, Berlin) and lysed in Fastprep Lysing Matrix B (MP Bio) for 15 s at speed 4. Clarified lysates (20 A_260_ units) were digested with 2000 U micrococcal nuclease (New England Biolabs) for 1 h ($25^\circ \textrm{C}$, shaking at 14 500 rpm). Digests were stopped with EGTA (final concentration, 6 mM), immediately loaded onto 10–55% (w/v) sucrose density gradients freshly prepared in sucrose buffer (100 mM NH_4_Cl, 10 mM MgCl_2_, 5 mM CaCl_2_, 20 mM Tris-HCl, pH 8, 1mM chloramphenicol, 2 mM dithiothreitol) and centrifuged (35 000 rpm, 2.5 h, $4^\circ \textrm{C}$) in a Beckman Coulter Optima L-80 XP ultracentrifuge and SW 40 Ti rotor. Gradients were fractionated (Gradient Station *ip*, Biocomp) and the 70S monosome fraction (identified by following fraction A_260_) was immediately frozen in liquid N_2_. RNA was extracted from fractions or cell pellets for total RNA using hot phenol:chloroform:isoamyl alcohol or hot phenol, respectively, as described previously [52, 53]. Total RNA was digested with DNase I, depleted of rRNA (RiboZero Bacteria, Illumina) and fragmented (Ambion 10X RNA Fragmentation Reagent) according to the manufacturer’s instructions. Monosome RNA and fragmented total RNA was size-selected (26–34 nt) on gels as described previously [54].

#### Library preparation, sequencing and data deposition

Libraries were prepared by vertis Biotechnologie AG (Freising, Germany) using a Small RNA protocol without fragmentation and sequenced on a NextSeq500 instrument (high-output, 75 cycles) at the Core Unit SysMed at the University of Würzburg. The data has been deposited in the NCBI Gene Expression Omnibus (GSE131514).

### Public data retrieval

#### *Escherichia coli* K-12 MG1655

Published proteomics data [55] were obtained from Supplemental Table S9 of the cited manuscript. Cultures were grown at $37^\circ \textrm{C}$ in LB until they completed ten divisions in exponential state. In order to test the ability of the tools to detect novel sORFs, we retrieved an additional *E. coli* MG1655 dataset, distinct from our newly generated dataset. We retrieved published [20] Ribo-seq (SAMN10583712, SAMN10583713) dataset for bacteria grown at $37^\circ \textrm{C}$ in MOPS EZ Rich Defined media with 0.2% glucose to an OD_600_ of 0.3. Experimentally verified novel sORFs were retrieved from Table 1 of the publication.

#### *Listeria monocytogenes* EDG-e

For *L. monocytogenes*, we utilized data from a published screen for antibiotic-responsive ribo-regulators [56]. We retrieved the Ribo-seq (SAMEA3864955) and RNA-seq (SAMEA3864956) datasets for the wild-type strain EDG-e from SRA. Cells were grown in brain heart infusion (BHI) medium at $37^\circ \textrm{C}$ to an OD_600_ of 0.4–0.5. The culture was supplemented with control medium for 15 min before harvesting. For our analysis, the untreated control library was used. Published proteomics data [57] were obtained from Supplemental Tables S2, S3, S4, S5, S6, S7, S8 of the cited manuscript. Cultures were grown at $37^\circ \textrm{C}$ to an OD_600_ of 1.

#### Pseudomonas aeruginosa PAO1

The data for *P. aeruginosa* is from a study investigating expression differences in strains with high sequence similarity but differences in substrate consumption efficiency using a multi-omics approach [58]. We retrieved the Ribo-seq and RNA-seq (SAMN06617371) datasets for the PAO1 wild-type strain grown on n-alkanes to mid-log phase. Corresponding proteomics data was retrieved from Supplemental Tables S21–S24 of the same publication.

#### Salmonella typhimurium 14028s

Finally, we used data generated to investigate the impact of the RNA-binding protein CsrA on *S.* typhimurium virulence-associated stress responses and metabolism [59]. We retrieved Ribo-seq (SRX3456030) and RNA-seq (SRX3456038) datasets for wild-type strain 14028s grown in LB medium at $37^\circ \textrm{C}$ to an OD_600_ of 0.5. The published [60] MS data were obtained from Supplemental Table S1 of the cited manuscript. Cultures were cultivated under identical conditions as for Ribo-seq.

### Bioinformatic analysis

We used part of a pre-release version of our HRIBO (high-throughput annotation by Ribo-seq) workflow, which we have developed to analyze prokaryotic ribosome profiling experiments [16, 35], to process Ribo-seq data prior to benchmarking. The genomes and annotations of *E. coli* K-12. MG1655 (ASM584v2), *L. monocytogenes* EGD-e (ASM19603v1), *P. aeruginosa* PAO1 (ASM676v1, ASM75657v1) and *S.* typhimurium 14028s (ASM2216v1) retrieved from the National Center for Biotechnology Information (NCBI) [61] were used. The HRIBO workflow consists of three steps: the preprocessing of the input data, the execution of the individual prediction tools, and a postprocessing step. A detailed description of how to run the RiboReport pipeline is provided in the RiboReport GitHub repository. To integrate the prediction tools into our pipeline, we created docker containers for each tool that were not available via bioconda [62]. The individual steps of the RiboReport pipeline are described in the following paragraphs.

#### Preprocessing

To generate the required input files for the benchmarking tools, adapters (see Supplemental Section F- Adapter sequences used for trimming) were first trimmed from the input reads using cutadapt [63]. Next, reads were mapped to the genome using segemehl [64], which has higher sensitivity than other mappers, and its high computational costs are still acceptable for small genomes. Finally, the reads mapping to ribosomal RNA or multiple genomic locations were filtered out using samtools [65]. Adapted annotation files were also generated, as several tools require very specific formatting of *gene transfer format (GTF)* files. DeepRibo requires coverage files as an input. The coverage files were produced using a custom-made script, following the instructions in the DeepRibo documentation [41]. In summary, we generated read alignments to the respective reference genomes for Ribo-seq and RNA-seq libraries in *BAM* (binary version of sequence alignment map format) as well as transcript files in *BED* (Browser Extensible Data) and read coverage files in *BEDGRAPH* format. In addition, we monitored the quality of each of these steps using fastQC and aggregated the results into a MultiQC [66] report.

#### Execution of ORF detection tools

Tools compatible with bacterial data and annotations were investigated: Ribo-TISH, REPARATION_blast, DeepRibo, SPECtre, IRSOM, ribotricer, PRICE, and smORFer. As we discovered that most tools designed for eukaryotes do not work (or less reliably) with reference annotations from NCBI [61], we chose to generate annotation files from our NCBI annotation in the older general feature format (v2 *GTF*), like those available from Ensembl Bacteria [67]. These files contain some features like transcripts and exons that are usually required for most eukaryotic tools, but which are not present in most general feature format (v3 *GFF*) files for bacteria. We chose to generate our own files instead of using the files from Ensembl Bacteria directly, as they were from different assemblies and would have introduced some bias. Since all tools, with the exception of Ribo-TISH, do not handle replicates, we selected a single replicate for each organism. Ribo-TISH was called using default parameters using the mapping files generated from the Ribo-seq data, the reference genome and the adapted annotation file. REPARATION_blast was run using default parameters with the Ribo-seq mapping files, the reference genome and annotation and the uniprot_sprot [68] database. Since REPARATION uses the commercial tool ublast internally, we replaced ublast with *protein blast* (blastp) [69] and adapted the tool to allow the input of *BAM* files. Since blastx is more sensitive while consuming more CPU-time compared with ublast [70], we expect that our modified tool behaves similarly in comparison to the original version. We made this adapted version, called REPARATION_blast, available via *bioconda* [62]. SPECtre was executed with default parameters, using a isoforms file created by cufflinks [71].

For DeepRibo, parameters for noise reduction need to be adapted for each dataset. We used the script provided in the DeepRibo GitHub repository (*s_curve_cutoff_ estimation.R*) for this purpose. This script provides cut-off values for *coverage* and RPKM (reads per kilobase million). Furthermore, we provided it with the requested input coverage and acceptor site coverage files, as well as the reference annotation, the reference genome, and the included pretrained model. IRSOM was called using default parameters and the included pretrained model for *E. coli*. All other pretrained models are dedicated to the use of eukaryotic organisms. Further, we used cufflinks to extract transcript regions from the alignment files generated from RNA-seq data and provide these to IRSOM for prediction. For ribotricer, we used a script provided in their GitHub repository to learn a phase-score cutoff using a Ribo-seq and RNA-seq library from our used datasets. This is important due to the difference in cutoff values between eukaryotic and prokaryotic data. Then, we created a ribotricer index file using our Ensembl-like annotation and the respective genome file. These files were then used to run ribotricer. For PRICE, we generated a genome index file with the script provided in their GitHub repository, our Ensembl-like annotation, and the respective genome file. For smORFer, we manually (not using our pipeline) created calibrated alignment files for *E. coli* as was described in the smORFer documentation. This was not possible for the other datasets due too high memory consumption. For these datasets we used a helper script, provided in the smORFer GitHub repository, to create calibrated alignment files using the middle nucleotide of each mapped read. These files were also recommended for TIS prediction and we therefore generated them for the novel sORF analysis as well. For *S.* typhimurium, the step for filtering the initial candidates for sequence periodicity using Fourier Transform failed, and we had to run the analysis without this step. For the TIS analysis of smORFer, we created a script to retrieve the next in-frame stop codon for each predicted start codon. This was done because we could not see the full potential of the tool due to a low coverage Ribo-seq library. Moreover, we tried to create two sets of smORFer predictions for each datasets. For one, we used the default length settings and for the second we increased the maximum ORF length to 3000 nt. We tested multiple upper boundaries, but the runtime and memory usage increased drastically with the change of this parameter. As we test for annotated ORFs, we had to increase the upper ORF length boundary in order for smORFer to be able to detect annotated long ORFs.

#### Postprocessing

Postprocessing steps were performed by parsing the prediction results of each tool into a *GTF* format file that can be used for evaluation. As each tool has a different output format, each result file had to be parsed differently. For ribotricer, REPARATION_blast, and SPECtre, we converted the results from a text file into *GFF* format. For Ribo-TISH, we used the *RiboPStatus* column to select only the best result for each start codon. For DeepRibo we used the *SS_pred_rank* column to select only the best result for each stop site. Finally, for IRSOM, which reports whether a result is coding or noncoding, we only used results labeled as coding. For PRICE we used both the filtered and unfiltered results. We transformed the final output tables into *GFF* format. As there were few results in the filtered file, we chose to use all predictions, as PRICE is predicting many truncated ORFs and otherwise cannot compete with the other tools. For smORFer, we transformed the output *BED* files (or *BED*-like tables) into *GFF* format. Additionally, the workflow generates multiple excel files containing different measures, like translational efficiency, RPKM, amino acid count and others. These files were used in order to assist with the manually labeled dataset of the annotated features.

#### Processing of MS data

MS data were first converted to *GFF* format. The exact steps required for the different datasets can be reproduced as described in the RiboReport proteomics directory.

### Benchmark of ORF detection

#### Manual labeling of translated regions based on Ribo-seq data

We tested the predictive power of the tools using ORFs within the NCBI annotation for each organism, which were labeled as translated or not based on inspection of paired Ribo-seq and RNA-seq libraries. For this, a human expert (S.L.S.) made judgments about whether each annotated ORF is `translated' or `not translated' as follows. Briefly, one RNA-seq replicate and its corresponding Ribo-seq (70S footprint) library (normalized to the lowest number of reads between the two) was loaded into the Integrated Genome Browser [72] together with the genome reference sequence and ORF annotation. RNA-seq and Ribo-seq coverage for each ORF was visually inspected at the same scale without knowledge of the locus tag or gene product name. Each experiment (organism) was curated independently. A single strand was labeled in one sitting. ORFs were called as ‘translated’ using the following criteria. First, coverage in RNA-seq and Ribo-seq libraries was required to be, generally, at least ten reads per nucleotide normalized by sample size. Due to uneven coverage across most ORFs, this was only a rough estimation. We therefore also discarded any positively labeled ORF with RPKM <1 as ‘not translated/expressed’ after the curation process. Second, the Ribo-seq signal was generally required to be comparable to the transcriptome library (i.e. TE approx. 1). Third, the shape of the Ribo-seq coverage over the ORF was considered: ORFs with Ribo-seq coverage near the start codon and/or restricted within ORF boundaries (and excluded from 5’/3’UTRs) were called as translated, even if the TE was <1. For manual labeling of the 33 western blot-validated sORFs from [20], the same approach was taken, with the exception that only the Ribo-seq library was inspected as no RNA-seq library was provided with the dataset. The associated TIS library is only included in screenshots and was not used for the manual labeling.

#### Computation of prediction quality

For each organism, we used the manually labeled datasets (*labels.gff*) to split the ORFs into two files (*positive_labels.gff*, *negative_labels.gff*) representing translated and nontranslated ORFs, respectively. The set of condition-positive ORFs (those labeled as translated in our manual curation) should therefore be found by a prediction tool, while the condition-negative ORFs (those labeled as not translated) and should not be called as translated).

To determine whether a prediction should be assigned to an annotated ORF from our benchmark set, we defined different overlap thresholds between the genomic coordinates of a prediction and the ORFs labeled as translated or nontranslated. The overlap was computed using bedtools intersect [73].

We set reciprocal overlap thresholds of 1%, 70% and 90%, requiring that the label–prediction overlap, and vice versa, is at least as big as the selected threshold. For example, the overlap threshold of 1% tests whether a tool detects translation at a certain locus at all, whereas the 90% threshold tests if a tool can also predict its correct length. The results created with a threshold of 1% are not a useful measure of a tool’s predictive performance, as this only reports whether a tool makes any prediction in the proximity of an ORF. We decided to use a threshold of 70% to emulate the inspection strategy of a researcher who will inspect ORFs of interest afterwards. This cutoff tests for translation of a locus but includes the possibility to identify novel truncated or nested ORFs.

Based on the intersection between the tool predictions and our manually labeled ORF sets, each ORF prediction was classified as a true positive (TP), true negative (TN), false positive (FP) or false negative (FN). An annotated gene with a positive label was counted as a TP if there was at least one prediction that was associated with the gene, and as a FN if no prediction was associated with the gene. An annotated gene with a negative label was counted as an FP if there was at least one prediction associated with the gene or a TN if no prediction was associated with the gene. The association of predictions and genes was determined for each tool and dataset individually. There were two cases where a prediction was not counted for a labeled gene. First, an annotated gene might have an overlap with multiple predictions from a given tool. In this case, only the prediction with the best predictive score or probability, depending on the tool, was considered. All other predictions were counted as suboptimals and ignored for the remaining analysis. Second, there were predictions that did not overlap with any annotated ORFs. These predictions were not counted at all, as the ground truth is not known in this case (i.e. we cannot determine whether they were novel predictions or FPs).

In addition to comparing the tools for the *E. coli* NCBI ORF annotation, we also investigated their performance on novel sORFs using a Ribo-seq dataset for *E. coli* that was generated in parallel with a TIS library that revealed 33 novel sORFs that were independently validated by western blotting (see subsection *Novel sORFs*).

To measure the prediction quality of the tools in determining the correct labels for each ORF of our benchmark, we computed the sensitivity and specificity of their predictions. Since our positive and negative datasets were unbalanced, we computed the F1 measure as an unbiased tool performance measurement. Furthermore, we plotted Precision–Recall Curves (PRCs) and calculated their area under the curve (AUC) to compare the performance of the different tools between the organism. The PRC avoids an overlap threshold bias, unlike the F1 measure, which can only be calculated for one overlap threshold. To compute PRCs, the positively and negatively labeled ORFs were used to generate the positive and negative datasets, respectively. Since the computed scores of the tools were not directly comparable, all predictions were ranked based on their given scores. Annotated ORFs without an associated prediction (FN and TN) were included in the ranking with the lowest possible score that each tool could provide.

Evaluation scripts are located in the evaluation directory of the RiboReport repository, with a description on how they were executed. The PRC and AUC were computed using scikit-learn [74] and plotted using matplotlib [75]. In addition to the PRC, each plot includes a baseline [$ \mathtt{baseline} = \mathtt{positive labels}/ (\mathtt{positive labels} + \mathtt{negative labels})$], which represents how many positive predictions are expected to occur by chance. For each Venn diagram, overlap sets of the correctly discovered, positively labeled ORFs were computed. We used the Jvenn webserver to produce the Venn diagrams [76] in Figures 4 and 6 and python scripts utilizing the seaborn [77] and simple_venn library for Figure S1.

#### Selection of subsets

Besides the whole *translatome* dataset, we also tested tool performance on the following subsets: (1) *close-proximity genes* were defined as groups or intervals of neighboring genes on the same strand with an intergenic distance of less than 200 nucleotides (https://github.com/RickGelhausen/RiboReport#extract-operon-regions-from-the-annotation). (2) *Stand-alone ORFs* are those that do not overlap with the *close-proximity gene* intervals. (3) *Small ORFs* were defined as genes with length $\leq 150$ nt (50 aa) [20]. Based on these definitions, we generated labeled positive (translated) and negative (not translated) sets for each subset. These files are available in our GitHub repository.

#### Computation of run time and peak memory consumption

Runtime and memory consumption of the tools was evaluated by running them individually on our newly generated *E. coli* dataset with either a single or with ten CPU threads. This analysis was run on a cloud instance using 28 VCPUs of an AMD EPYC (with IBPB) processor and 64 GB of RAM. The used operating system was Ubuntu 20.04.3 LTS (kernel version 5.4.0-88-generic).

**Table 2:** **Generation of a curated benchmark ORF set**. The benchmark set archives contain *GFF* files for labels of all annotated ORF sets (positive/negative), MS labels, tool predictions, close-proximity genes, genome sequences, and reference annotations to enable inspection in a genome browser. Links to the original data sources are provided. For each dataset the sequencing depth is given (total number of reads times average read length divided by genome length) [80]. The number of ORFs from each annotated ORF set (*translatome*, *sORFs*, *close-proximity genes* and *stand-alone genes*) that have been identified as translated (positive) or nontranslated (negative) are listed.

Organism	*E. coli*		*L. monocytogenes* [56]		*P. aeruginosa* [59]		*S.* typhimurium [58]	
Benchmark set [zip]	*E. coli*		*L. monocytogenes*		*P. aeruginosa*		*S.* typhimurium	
Growth conditions	WT, LB @ 37$^{\circ }$C		WT, BHI @ 37$^{\circ }$C		WT, n-alkanes		WT, LB @ 37$^{\circ }$C	
Data	GSE131514		SAMEA3864955		SAMN06617371		SRX3456030	
			SAMEA3864956				SRX3456038	
Sequencing depth	42.98		939.76		81.92		38.92	
Set	Positive	Negative	Positive	Negative	Positive	Negative	Positive	Negative
Translatome	2763 (65%)	1485 (35%)	2288 (80%)	579 (20%)	3935 (71%)	1638 (29%)	3284 (66%)	1689 (34%)
sORFs	54 (48%)	60 (52%)	7 (100%)	0 (0%)	7 (58%)	5 (42%)	31 (31%)	69 (69%)
Close-proximity genes	1794 (64%)	1015 (36%)	1622 (80%)	432 (20%)	2511 (69%)	1113 (31%)	1947(66%)	1010(34%)
Stand-alone genes	969 (67%)	470 (33%)	666 (82%)	147 (18%)	1424 (73%)	525 (27%)	1337 (66%)	679 (34%)

#### Evaluation of manual labeling with MS data

To validate our labeling method, each annotated ORF in the four bacterial genomes was first manually labeled as translated or not based on manual inspection of Ribo-seq data in a genome browser (see above section on manual labeling for details). We then validated our labeling approach by comparison to available published MS datasets (proteomics) for the same strains grown under similar conditions (see Supplemental Section—Validation of labeling method, Figure S1). The MS data were selected to be as similar as possible to the Ribo-seq experimental conditions see above section, (Data Retrieval).

## 3 Results & Discussion

### Applicability of available tools to bacterial data

By screening reviews [47, 78] and recently published studies [38, 39, 41, 42, 44], we found 12 stand-alone Ribo-seq based ORF detection tools (Table 1). Additionally, we identified several tools that predict potential ORFs from only RNA-seq (transcriptome) data and included the newest example (IRSOM) for comparison. We first tested these thirteen tools for their compatibility with bacterial annotations using our *E. coli* benchmark dataset. We found that only eight tools could accept and process this dataset: REPARATION_blast, Ribo-TISH, IRSOM, SPECtre, smORFer, PRICE, ribotricer, and DeepRibo. Since RiboTaper and RiboHMM do not work with bacterial annotations, we could not run them. We were not able to install Rp-Bp on our cluster system or locally in a reasonable amount of time. For ORFrater and RibORF, several steps of their pipelines could be executed, but we did not obtain a result output. Seven of the tools that could handle bacterial data are open source. However, REPARATION uses the proprietary homology search tool ublast [79] internally, which we replaced by the open tool blastp [69] to make the tool viable for open source usage, e.g. in pipelines. We refer to this version as REPARATION_blast.

### Benchmark datasets

**Figure 2:**
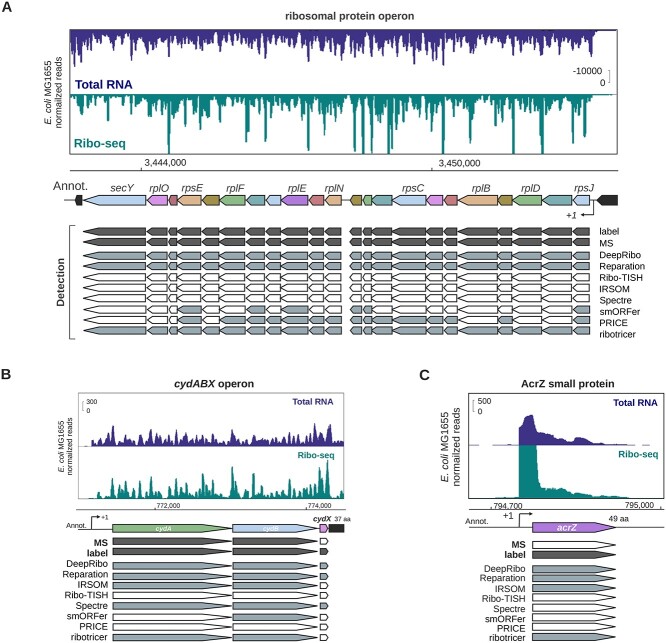
**Comparison of manual Ribo-seq curation, proteomics data, and tool performance for representative *E. coli* genes. for the translatome sets of the four organisms**. Highly conserved and translated long ribosomal protein operon between *rpmJ* and *secY*, including several essential sORFs. Related to Supplemental Figure S4. (B) The highly conserved Gammaproteobacteria *cydABX* operon in *E. coli*. The final gene in the operon, *cydX*, encodes a functional small protein [81]. Related to Supplemental Figure S2. The space between *cydA* and *cydB* is 15 nt. (C) The ORF encoding the small protein AcrZ, an antibiotic efflux pump specificity factor [82, 83]. For all screenshots, genes that are detected in the publicly available proteomics (MS) dataset and by manual curation of the Ribo-seq data (label) are indicated in dark gray. Detection by the indicated tools at a 70% overlap threshold is indicated in gray based on Ribo-seq data (or RNA-seq, IRSOM). Those that are not detected as translated are white. Transcriptional start sites, if available, are indicated with a bent arrow ($+1$).

A robust performance evaluation of sORF detection tools requires data from a variety of prokaryotic organisms. Therefore, we added several publicly available datasets covering different bacterial species to our *de novo*-generated *E. coli* benchmark set. Criteria for selection included quality [published, sufficient sequencing quality (e.g. the sequencing quality score or per base sequence content), sufficient documentation (i.e. adaptor sequences)] as well as the availability of a paired RNA-seq library to aid manual labeling of translation and for evaluation using the RNA-seq-based tool IRSOM. In total, the four benchmark datasets include our newly generated *E. coli* dataset and publicly available datasets for wild-type strains of *L. monocytogenes*, *P. aeruginosa* and *S.* typhimurium (Table 2) (see Materials and Methods for details). We used these data to manually label the translation status of all annotated ORFs in each genome (for details, see Materials and Methods). Labeling quality was assessed by comparison to MS data and inspection of specific examples (Figure 2A, Supplemental Figures S1–S3). These manually labeled Ribo-seq ORF sets are, to our knowledge, the first available for bacterial Ribo-seq data for the purpose of tool benchmarking and are available from the GitHub repository.

### Benchmark results

DeepRibo and REPARATION_blast have been recently compared for their performance [41]. However, this comparison was based on a dataset used to train the default model of DeepRibo; this is therefore not an unbiased benchmark. We thus used our novel, comprehensive benchmark set to evaluate the performance of all eight ORF detection tools that we found accept bacterial data (Table 1). Prediction quality metrics were computed (see Materials and Methods subsection Benchmark of ORF detection) for the whole *translatome*, as well as for specific ORF subsets that have properties that could possibly influence prediction results. We compared whether the tools show a different behaviour for ORFs of genes in close-proximity and stand-alone regions, as well as for annotated sORFs and a set of western blot validated novel sORFs from *E. coli* using an additional Ribo-seq dataset [20].

#### Bacterial tools generally show more robust performance

**Figure 3:**
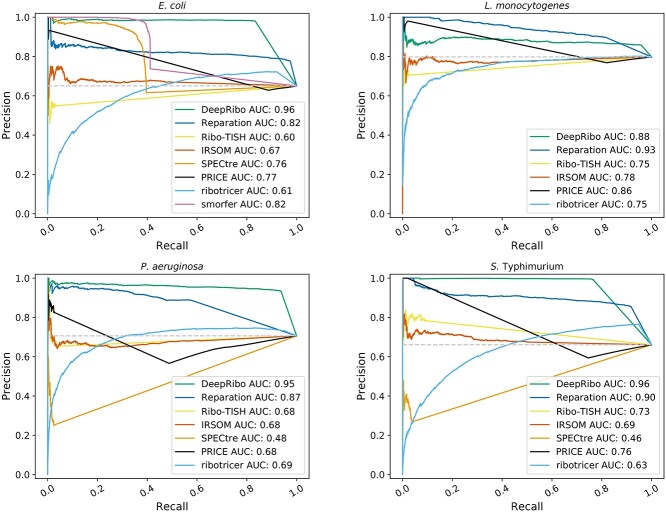
**PRCs for *E. coli*, *L. monocytogenes*, *P. aeruginosa* and *S.* typhimurium**. Predictions were ranked according to the score provided by each tool (e.g. the probability for REPARATION_blast or the prediction rank for DeepRibo). A prediction was associated with a labeled ORF if more than a 70% overlap existed between the sequence of the prediction and the labeled ORF. If a labeled ORF had no prediction overlapping more than 70% of its coding region it is classified as not predicted. The ranked instances were then used to plot the PRC and to calculate the AUC. The gray baseline indicates how many predictions are expected to occur by chance.

The tools were first compared on the whole complement of annotated ORFs for each organism (hereafter the *translatome* set) (Table 2). Tool performance was measured by determining the AUC of a PRC [84]. We selected this metric because the number of positively and negatively labeled ORFs were imbalanced, especially for *L. monocytogenes* (80% of ORFs were in the positive set). The PRC compares the recall of the tool against its precision value for a given score cutoff. The recall in this context is the fraction of correctly predicted, labeled ORFs (TPs) versus the sum of all positively labeled ORFs (including FN), yielding (TP/TP + FN). The precision is the fraction of correctly predicted, positively labeled ORFs (TPs) versus the sum of all positively predicted ORFs (including FP) yielding (TP/TP + FP). We compared the AUC for each tool at different overlap thresholds to test not only if they were able to predict the presence of an ORF, but also if they could correctly determine its length (Table 3). We used thresholds of 1%, 70%, and 90% (i.e. the prediction must cover at least 1%, 70%, 90% of the ORF length). For single-gene examples of TP, FP, TN, FN, please see Supplemental Figure S3. DeepRibo, REPARATION_blast, SPECtre, ribotricer, and IRSOM showed a stable performance over the three thresholds, meaning that when they predict an ORF they also can correctly predict its length. Ribo-TISH, smORFer, and PRICE, however, often predicted only a short region of the annotated ORF as translated. This can be observed, for example, in *E. coli*, where the high AUC of 0.85 for the 1% overlap threshold then drops to an AUC of 0.6 for the 70% overlap threshold. The PRCs for an overlap threshold of 70% (Figure 3) show that DeepRibo and REPARATION_blast performed well for detection of the *translatome* benchmark ORF sets from all four organisms (AUC > 0.8). smORFer also had a high AUC for the *E. coli* dataset, whereas it had a low number of TP predictions compared with the two other tools designed for bacteria. We could not run all datasets using smORFer as we ran out of memory for *L. monocytogenes* and *P. aeruginosa* when using a maximum ORF length of 3000 nt, due to the higher sequencing depth of these datasets (Table 2). As smORFer was designed for sORFs it would be unfair to use default settings, as it then would not be able to detect ORFs greater than 50 codons. ribotricer generally predicted many TPs, slightly more than DeepRibo, but also predicted more FPs than the other tools. In contrast, IRSOM, PRICE, SPECtre, and Ribo-TISH generally had substantially lower AUCs—almost close to random (gray baseline, see Methods subsection: Benchmark of ORF detection). PRICE tended to only predict truncated ORFs and thus only a few TPs for the 70% overlap threshold.

**Table 3:** **Overall tool performance at different overlap thresholds**. The AUC of the PRC is given for the tools with each of the four organism datasets (whole *translatome* ORF set) at the prediction overlap thresholds of 1%, 70% and 90%. The overlap threshold is the percentage of the ORF length that the prediction must satisfy.

Organism:	*E. coli* (AUC)			*L. monocytogenes* (AUC)			*P. aeruginosa* (AUC)			*S.* typhimurium (AUC)		
Overlap:	1%	70%	90%	1%	70%	90%	1%	70%	90%	1%	70%	90%
DeepRibo	0.97	0.96	0.95	0.88	0.88	0.88	0.95	0.95	0.95	0.97	0.96	0.95
REPARATION_blast	0.82	0.82	0.82	0.93	0.93	0.93	0.88	0.87	0.87	0.88	0.90	0.89
Ribo-TISH	0.85	0.60	0.60	0.83	0.75	0.75	0.85	0.68	0.65	0.87	0.73	0.73
IRSOM	0.67	0.67	0.67	0.78	0.78	0.78	0.68	0.68	0.68	0.68	0.69	0.69
SPECtre	0.76	0.76	0.76	—	—	—	0.48	0.48	0.48	0.46	0.46	0.46
smORFer	0.94	0.82	0.78	—	—	—	—	—	—	—	—	—
PRICE	0.57	0.77	0.77	0.74	0.86	0.86	0.6	0.68	0.71	0.62	0.76	0.77
ribotricer	0.61	0.61	0.61	0.75	0.75	0.75	0.69	0.69	0.69	0.62	0.63	0.63

**Figure 4:**
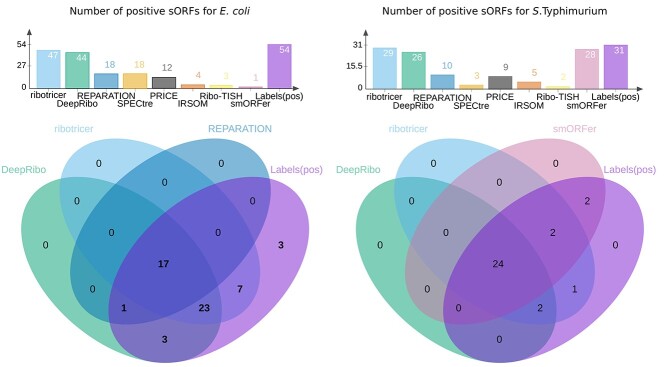
**Comparison of the correctly detected sORFs for *E. coli* and *S.* typhimurium by each tool to manual labeling** Top: the number of correctly predicted, translated sORFs by DeepRibo, REPARATION_blast, Ribo-TISH, IRSOM, SPECtre, ribotricer, PRICE, smORFer or manual labeling. Bottom: the overlap of sORFs detected by DeepRibo (green), REPARATION_blast (blue), smORFer (reddish purple) or ribotricer (sky blue) with the sORFs labeled as translated (purple) for *E. coli* (left) and *S.typhimurium* (right). Only the three tools that detected most sORFs are shown in the Venn diagram. The number of TP sORFs detected by the tools were determined at an overlap threshold of 70%.

DeepRibo showed the highest AUC values for *E. coli*, *S.* typhimurium, and *P. aeruginosa*, suggesting it has the highest predictive power for most organism datasets, whereas REPARATION_blast performed best for *L. monocytogenes*. A possible explanation for this is that the organisms DeepRibo was trained on might have different genomic characteristics compared with *L. monocytogenes*. However, it could also be the result of experimental differences that change the distribution of the read coverage. ribotricer had an average AUC as it also predicted many FPs. ribotricer learns a phase score cutoff based on Ribo-seq and RNA-seq libraries. This cutoff turns out to be very low for bacterial data. It might be that the automatic cutoff detection does not work well for prokaryotes, as it was designed for eukaryotic data. We next investigated the sensitivity, specificity and F1 measure of the tools (Table 4). The F1 measure, which is the harmonic mean of recall and precision, showed that IRSOM performed surprisingly well, even though it only relies on RNA-seq data. IRSOM, however, could not compete with the tools designed for bacterial Ribo-seq data (DeepRibo and REPARATION_blast). This same trend was observed for sensitivity and specificity. DeepRibo showed overall a strong predictive performance and was only outperformed by REPARATION_blast for the *L. monocytogenes* dataset. The lower AUC value in this case was due to a higher FP rate for this dataset (see Supplemental Tables 1–4). ribotricer was the only tool designed for eukaryotes that also performed very well for bacterial data. It had a similar F1 measure as DeepRibo and REPARATION_blast, although slightly lower. Furthermore, sensitivity and specificity measures were also comparable.

The sensitivity of Ribo-TISH was low for all four datasets (Table 4). As already seen for the AUC at different overlap thresholds (Table 3), Ribo-TISH did not predict ORFs precisely, but rather predicted a short signal nested in the region of a labeled ORF (average sensitivity for overlap threshold 1% was 0.6). SPECtre, similar to Ribo-TISH, had low sensitivity. However, its specificity, while comparable, was slightly lower. We could not generate SPECtre results for *L. monocytogenes* reproducibly within 72 h. The lower performance of Ribo-TISH and SPECtre might be explained by the fact that they were not specifically designed for bacteria, which have distinct translatome structures. In addition, both of these tools rely on three nucleotide periodicity, which is often not pronounced in bacterial datasets due to experimental issues [40]. Moreover, SPECtre depends on the transcript-calling performance of cufflinks [71], which means that it might also be affected by the quality of the coupled RNA-seq data. PRICE had a generally low F1 measure. This was likely caused by the prediction of many truncated ORFs that did not pass the overlap threshold. While PRICE offers an additional filtering method that reduces the amount of predictions, this did not change the percentage of truncated ORFs it predicts, still leaving a list of about 300 predictions. As smORFer is modular, it offers a variety of ways to conduct the analysis. We tried using the approach recommended for this tool, which included a calibrated alignment file to filter for the best start codons. We omitted the optional Fourier Transform step, as this further reduced the number of results. While this is desirable for novel ORF detection, it would reduce the AUC substantially (data not shown). smORFer was designed for detection of sORFs, which might explain its lower performance in predicting annotated ORFs, which are generally longer.

In addition to computation of global performance metrics, we also qualitatively compared how the tools performed for specific ORFs. We inspected coverage for specific examples of ORFs in genomic regions conserved between the four benchmark organisms and compared this with their detection by each of the five tools at a 70% overlap threshold. For this and future comparisons, genome browser tracks for all tool predictions can be found as prediction.gff files in the archives of each respective organism RiboReport repository (data/*/misc_*.zip, * = organism). We first compared the detection of genes in a ribosomal protein island with conserved synteny to assess our labeling performance vs MS, all of which are likely *bona fide*, translated ORFs under the conditions tested due to their central role in translation. Comparison of detection by the eight tested tools in all four organisms showed that in general, DeepRibo, REPARATION_blast, and ribotricer called these ORFs as translated (Figure 2A, Supplemental Figure S2). In comparison, SPECtre and Ribo-TISH did not detect any of the 22 ORFs at this threshold, while PRICE and smORFer detected only a handful. Surprisingly, RNA-seq based IRSOM was mildly successful, detecting a handful of ORFs in the organisms other than *E. coli*. We also examined tool predictions of two genes in an operon shared by all four bacteria: that encoding a terminal oxidase (*cydAB* in *E. coli*, *S.* typhimurium, and *L. monocytogenes*, *cioAB* in *P. aeruginosa*) (Figure 2B, Supplemental Figure S4). Both *cydA* and *cydB* were labeled as translated and detected by DeepRibo and ribotricer in all organisms, while REPARATION_blast detected all but *cioAB* in *P. aeruginosa*. The other tools showed variable detection of the *cydA*/*cydB* homologues, with PRICE detecting both genes in *P. aeruginosa* and *L. monocytogenes*. Ribo-TISH and smORFer did not detect either in any organism. Closer inspection of the Ribo-TISH predictions (data not shown) indicated that the tool was predicting several very short nested ORFs in *cydA* and *cydB*. Together, these comparisons of tool sensitivity and specificity on the whole translatome ORF sets for each of the four bacterial species shows that the bacterial Ribo-seq tools REPARATION_blast, ribotricer and DeepRibo are superior to smORFer and all other eukaryotic tools such as IRSOM and Ribo-TISH.

#### ORFs in close-proximity and stand-alone genes

A unique feature of bacterial genomes is the operon structure: several genes, often of related function, are transcribed as one polycistronic mRNA. Operons often have small distances between ORFs that might lead to ambiguity in associating Ribo-seq signal with neighboring ORFs. They might even include overlap of coding regions. These features could presumably affect ORF prediction tools. Therefore, we tested whether the predictive power of the tested tools is different for ORFs translated from genes having start/stop codon within 200 bp (close-proximity) compared with single transcribed genes (stand-alone), (Table 5).

**Table 4:** **Detailed tool performance measures for 70% overlap**. The sensitivity or true positive rate (TPR), specificity or true negative rate (TNR) and the F1 measure were calculated for each tool with each organism benchmark dataset (*translatome*). The sensitivity highlights how well the positive labels are detected and the specificity reveals how well negatively labeled ORFs are not predicted by the tools. The F1 measure is an unbiased tool accuracy measurement. The values were calculated with the requirement that the prediction of an ORF must be covered by at least 70% of its coding sequence

Organism	*E. coli*			*L. monocytogenes*			*P. aeruginosa*			*S.* typhimurium		
measure	TPR	TNR	F1	TPR	TNR	F1	TPR	TNR	F1	TPR	TNR	F1
DeepRibo	0.83	0.97	0.90	0.96	0.37	0.91	0.94	0.84	0.94	0.77	0.98	0.86
REPARATION_blast	0.98	0.48	0.86	0.82	0.63	0.85	0.59	0.82	0.7	0.92	0.69	0.88
Ribo-TISH	0.02	0.96	0.05	0.02	0.96	0.05	0.04	0.95	0.07	0.1	0.95	0.17
IRSOM	0.52	0.53	0.58	0.42	0.51	0.54	0.62	0.3	0.65	0.5	0.53	0.58
SPECtre	0.39	0.54	0.48	—	—	—	0.03	0.82	0.05	0.04	0.77	0.07
smORFer	0.41	0.73	0.53	—	—	—	—	—	—	—	—	—
PRICE	0.12	0.98	0.21	0.2	0.96	0.33	0.54	0.88	0.68	0.27	0.99	0.43
ribotricer	0.92	0.34	0.81	1	0.01	0.89	0.95	0.17	0.83	0.95	0.43	0.84

**Table 5:** **Prediction of ORFs from genes within close-proximity.** The predictive power of the eight tools for translation of genes either within close-proximity or stand-alone (alone) was compared via the AUC for PRCs computed using an overlap threshold of 70%.

Organism	*E. coli* (AUC)		*L. monocytogenes* (AUC)		*P. aeruginosa* (AUC)		*S.* typhimurium (AUC)	
ORF type	close-proximity	alone	close-proximity	alone	close-proximity	alone	close-proximity	alone
DeepRibo	0.96	0.96	0.88	0.91	0.95	0.95	0.96	0.96
REPARATION_blast	0.82	0.82	0.93	0.95	0.88	0.89	0.88	0.93
Ribo-TISH	0.59	0.62	0.75	0.77	0.73	0.71	0.71	0.74
IRSOM	0.65	0.71	0.78	0.83	0.66	0.71	0.65	0.74
SPECtre	0.74	0.8	—	—	0.43	0.57	0.43	0.73
smORFer	0.81	0.84	—	—	—	—	—	—
PRICE	0.75	0.81	0.86	0.89	0.66	0.7	0.75	0.76
ribotricer	0.6	0.36	0.75	0.77	0.68	0.71	0.59	0.69

We classified the annotated ORFs of each of the four organisms as originating from genes in close-proximity or stand-alone (see Materials and Methods, Selection of subsets). We then calculated the AUC of PRCs calculated at a overlap threshold of 70% for all eight tools with either the *close-proximity* or *stand-alone* gene sets separately for each organism (Table 5). DeepRibo  ribotricer and REPARATION_blast had similar or better performance for ORFs of close-proximity genes compared to the other tools (with the exception of the *Listeria* dataset). The other tools performed worse in all benchmark sets for genes located in operons compared with single-standing genes, which indicated a clear advantage of tools designed for bacteria in this regard, with the exception of ribotricer that performed equally well, while having more false positive predictions.

Above, we found that the bacterial tools DeepRibo and REPARATION_blast were able to detect most ORFs in a highly conserved ribosomal protein operon and *cydAB*/*cioAB* terminal oxidase operons (Figure 2A & 2B, Supplemental Figures S2 and S4A–C), whereas the other tools performed less well. Interestingly, *cydA* and *cydB* from *L. monocytogenes* overlap by 14 nt and were detected poorly by both IRSOM and Ribo-TISH (Supplemental Figure S4C). We selected an additional, more weakly expressed eight-gene operon (*ydjX, ydjY, ydjZ, ynjA, ynjB, ynjC, ynjD, ynjE*) in our *E. coli* dataset for inspection (Supplemental Figure S5A). Here, all genes were detected by IRSOM, and only some were missed by REPARATION_blast and ribotricer. The remaining tools performed poorly, including DeepRibo, possibly because it has a more stringent expression cutoff. None of these genes were manually labeled as translated because of their overall low signal in both Ribo-seq and RNA-seq libraries. In addition, we also inspected the well-characterized overlapping ORFs *btuB* and *murI*, which share 56 bp at the 3’ end of *btuB*, in our *E. coli* dataset. All of the tools except Ribo-TISH, SPECtre, and PRICE called both ORFs as translated (Supplemental Figure S5B). Finally, we inspected an example of a leaderless ORF, *rluC*, in the *E. coli* dataset (Supplemental Figure S5C). The same five out of the eight tools detected *rluC* translation. Together, our global and single-locus observations suggest that the bacterial tools perform relatively well for both single-standing and operon-encoded genes.

#### High sensitivity comes with high false positive rate in predicting sORFs

Genome annotations are notorious for lacking sORFs - those encoding proteins of 50 aa or less [1]. We therefore tested the performance of the tools solely on short genes by constructing a subset for each of the four organisms including only annotated ORFs of 50 codons or less. The general incompleteness of sORF annotation in bacteria is supported by the *L. monocytogenes* (2.9 Mbp) and *P. aeruginosa* (6.3 Mbp) *sORF* sets, which were smaller (seven and 12 sORFs, respectively; Table 2) than might be expected based on their genome size compared with *E. coli* (4.6 Mbp, 114 sORFs) and *S.* typhimurium (5.1 Mbp, 100 sORFs), which are considered some of the best annotated organisms for sORFs [15]. We therefore exclusively investigated the *E. coli* and *S. typhimurium sORF* sets, which were large enough for unbiased investigation.

Our manual labeling of the *E. coli* and *S*. *typhimurium sORF* subsets suggested that 54 of 114 and 31 of 100 sORFs, respectively, were translated under the investigated condition (Figure 4, top graphs and Table 2). Inspection of the tool predictions showed that ribotricer detected 47, DeepRibo 44, SPECtre 18 and REPARATION_blast 18 of the 54 positively labeled sORFs in the *E. coli sORF* set (Supplemental Table 13). For *S.* typhimurium, ribotricer flagged 29 of 31 positively labeled sORFs as translated, whereas smORFer and DeepRibo flagged 28 and 26, respectively (Figure 4, top). In contrast, IRSOM and Ribo-TISH detected hardly any of the positively labeled sORFs in these organisms (4/3 out of 55 for *E. coli* and 5/3 out of 31 for *S.* typhimurium, respectively). This shows that these tools do not perform well for sORF discovery in bacteria. All 18 sORFs detected by REPARATION_blast in *E. coli* were also detected by DeepRibo (Figure 4, bottom left). ribotricer detected seven sORFs that were not detected by the other tools and has the overall best performance in detecting sORFs for our chosen datasets. This was unexpected, as ribotricer was developed in eukaryotes. ribotricer, DeepRibo and REPARATION_blast made only a few false positive sORF predictions for *E. coli* and *S.* typhimurium (8/7/9 and 11/5/1, respectively) and correctly did not predict most of the sORFs that were labeled as not translated (52 out of 53) (Supplemental Table 13). Our data suggest that ribotricer and DeepRibo work well for detecting sORFs, since they detect nearly all annotated examples in both datasets. smORFer detected most positively labeled *S.* typhimurium sORFs, but only one *E. coli* sORF (Figure 4). We tried filtering with both manually calibrated alignment files and automatically generated middle nucleotide alignment files, but this did not change the number of sORFs predicted. We investigated whether the read count cutoff was to blame, but both datasets should have sufficient read coverage. For *S.* typhimurium, we did not filter for sequence periodicity, which left us with slightly more than 45 000 results. This was likely the cause of the high proportion of sORFs correctly detected by smORFer for this dataset. ribotricer performed well and tended to predict more sORFs correctly, while sharing a large overlap with the predictions of the other tools, as can be observed for *S.* typhimurium (Figure 4, bottom right). We assume that one of the main problems for smORFer was the detection of the correct start codons based on the Ribo-seq library alone. This problem would likely be solved by using a TIS library as described in their publication [44]. Three positively labeled *E. coli* sORFs were not detected by any of the tools (Figure 4).

We next inspected specific examples of positively labeled sORFs for their coverage compared with their tool predictions. Translation of the ORF encoding the *E. coli* small membrane protein AcrZ (49 aa), a regulatory component of the AcrB-TolC antibiotic efflux pump [15], was detected by DeepRibo, REPARATION_blast, ribotricer and even IRSOM via RNA-seq coverage, but not Ribo-TISH (Figure 2C). SgrT, encoded by the dual function sRNA SgrS [15], was identified as translated by DeepRibo and REPARATION_blast (Supplemental Figure S5D). Again, we revisited the *cydAB*/*cioAB* operons (Supplemental Figure S4). In many proteobacteria, a small protein component of the terminal oxidase complex is encoded downstream of *cydAB*/*cioAB* [85]. For example, CydX (37 aa) of *E. coli* and *S.* typhimurium is encoded downstream of *cydB*, whereas the putative sORF *cioZ* is encoded downstream of *P. aeruginosa* CioB (Figure 2B, Supplemental Figure S4A and B). All three of these sORFs were manually labeled as translated in *E. coli*, *S*. *typhimurium* and *P. aeruginosa*. At an overlap threshold of 70%, DeepRibo also detected translation of all three sORFs, whereas REPARATION_blast only detected the enterobacterial sORFs and SPECtre detected only *E. coli cydX*. IRSOM and Ribo-TISH did not call any of the sORFs as translated. So far, a similar small protein has not been detected in Firmicutes such as *L. monocytogenes* [85]. We therefore also inspected a different validated sORF from *L. monocytogenes*, since it does not encode a *cydX*. The sORF *lmo1980* [57] was labeled manually as translated and also detected only by the bacterial ORF prediction tools DeepRibo and REPARATION_blast (Supplemental Figure S4D).

#### Novel *E. coli* sORFs

Up to this point, we focused only on previously annotated ORFs. However, the discovery of novel sORFs is one of the most interesting applications of Ribo-seq [30]. To understand how well the different tools can detect novel, potentially more challenging, sORFs, we also ran our benchmark pipeline on the untreated (no retapamulin) Ribo-seq library that was generated as part of a TIS profiling experiment to experimentally identify novel *E. coli* sORFs [20]. This study validated the translation of 33 new sORFs detected by TIS profiling by epitope tagging and western blotting. Thirty-one of these 33 ORFs meet our definition of an sORF ($\leq 50$ aa). We labeled these 31 sORFs based on Ribo-seq coverage alone (no RNA-seq library was available and TIS coverage was not used) without knowledge of western blot results. This suggested that 19 of the 31 sORFs showed significant Ribo-seq coverage and are likely translated. We then compared the output of DeepRibo, REPARATION_blast, Ribo-TISH, ribotricer, and PRICE to detect how many of the 19 positively labeled novel sORFs where predicted by each tool. As ribotricer needs an RNA-seq library to determine the best phase score cutoff, but not for the prediction process itself, we chose a very low cutoff based on our observations for the four benchmarking datasets. We did not include SPECtre or IRSOM in this analysis, since these tools require an RNA-seq library, which was not available. However, since SPECtre did not predict any ORFs outside of the existing annotation for the other benchmark datasets (Supplemental Tables S1, S3 and S4), this suggests it likely has very limited utility in the identification of novels ORFs in bacteria. Inspection of the predictions for the remaining six tools showed that REPARATION_blast, ribotricer, PRICE and Ribo-TISH did not detect any of the 31 novel sORFs (Supplemental Table S14). These tools were then omitted from the comparison. In total, DeepRibo predicted 18 478 potential novel sORFs. Considering that only ~4000 ORFs (of which 114 are sORFs) are currently annotated in *E. coli*, many of these predictions are likely false positives.

**Table 6:** **Detection of novel *E. coli* sORFs by DeepRibo and smORFer**. Successfully predicted, experimentally verified novel sORFs [20] with their score and rank for all novel sORF predictions. For smORFer, the rank is based on the TIS read counts (RPF). Entries marked with X indicate missed predictions due to low Ribo-seq or TIS coverage

Gene name	smORFer Rank	smORFer RPF	DeepRibo Rank	DeepRibo Score
*ysaE*	73	1718	519	−2.111
*ysgD*	89	1452	115	−1.115
*ydgV*	183	612	X	X
*ychT*	292	334	42	−0.464
*yncP*	414	169	174	−1.464
*ynaN*	472	111	427	−2.000
*yqgH*	485	97	X	X
*ythB*	489	83	23	0.006
*yhgP*	492	90	X	X
*argL*	495	87	X	X
*yhiY*	515	67	X	X
*ybgV*	516	66	X	X
*yibX-S*	516	66	X	X
*ytiB*	539	43	1,129	−2.613
*yljB*	544	38	759	−2.353
*ytgA*	546	36	X	X
*yfiS*	549	33	61	−0.600
*ysdE*	552	30	X	X
*yriB*	555	27	X	X
*ykiE*	561	21	45	−0.481
*evgL*	556	26	X	X
*ybiE*	571	11	688	−2.282
*yicU*	572	10	X	X
*yqhJ*	573	9	5,352	−4.078
*yecV*	573	9	X	X
*yqgG*	576	6	15	0.169
*yadX*	576	6	498	−2.080
*ymiD*	577	5	46	−0.491
*yqiM*	X	X	520	−2.112
*yodE*	X	X	26	−0.006
*yriA*	X	X	X	X

**Figure 5:**
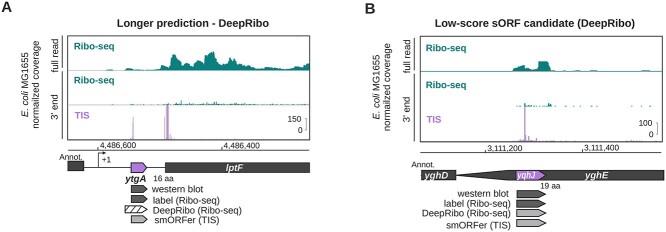
**Detection of novel, western blot-validated *E. coli* sORFs previously discovered by TIS profiling based.** The translation of 31 western blot-validated sORFs previously identified and validated in *E. coli* [20] was labeled by manual curation of the same Ribo-seq data. Labels were compared with DeepRibo predictions based on the Ribo-seq data at an overlap threshold of 70% based on Ribo-seq data (DeepRibo) or TIS data (smORFer). (A) The sORF *ytgA* (16 aa), in the 5’UTR of *lptF*, was labeled as translated and detected in TIS data by smORFer, but the DeepRibo prediction is extended at the 5’ end by three codons. (B) The low-ranked (DeepRibo) sORF *yqhJ* (19 aa). The ORF was detected by DeepRibo based on Ribo-seq data with a score of -4.078, as well as by TIS profiling by smORFer. Dark gray genes were detected by western blot or labeling. Gray genes were detected by the tools. Hatched arrows were detected, but with a slightly different length or position. White ORFs were not detected. The TIS track was not used for manual curation or DeepRibo predictions and was only used for smORFer. Transcriptional start sites, if available, are indicated with a bent arrow ($+1$).

**Figure 6:**
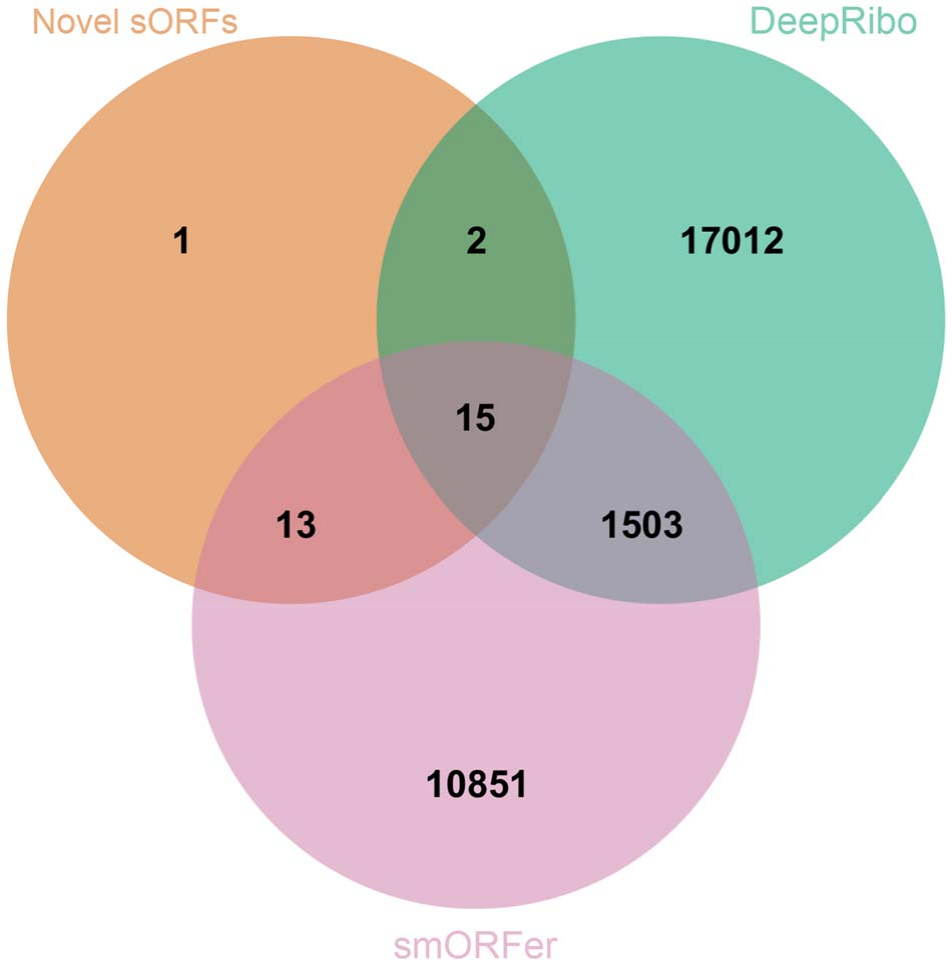
**Overlap of novel sORFs detected by DeepRibo and smORFer and a set of experimentally verified sORFs from a published *E. coli* dataset**. All predicted novel sORFs for DeepRibo and smORFer were compared with 31 novel sORFs recently detected by TIS profiling and verified by western blot in *E. coli* (orange) [20]. For DeepRibo only the Ribo-seq library was used and for smORFer only the TIS library was used.

DeepRibo provides a score for each detected ORF (novel and annotated), where ORFs with a higher score are of higher confidence. This score was can be used to generate a ranking. However, it is left to the user to find an appropriate cutoff. We found that DeepRibo predicted 17 of the 31 verified novel sORFs with no cutoff applied (Figure 6). To simulate the selection of novel sORFs for experimental verification, we filtered for the top 100 predicted by DeepRibo. Seven of these predicted novel sORFs [excluding *ynfU* (56 aa), *yibX* (80 aa)] were previously identified by TIS profiling and validated by western blotting [20] (Table 6). The next seven validated sORFs from this study are then among the top 520 predictions, which would already be a large number for manual inspection or experimental evaluation. We therefore recommend manual inspection of Ribo-seq coverage of the top 100 sORFs, which is manageable, followed by western blot validation of a handful including candidates for downstream functional characterization. Alternatively, the top 500 could be reinvestigated using available expression or functional genomics datasets to prioritize those that might represent true sORFs that encode small proteins with interesting functions, as was performed previously for *S*. *typhimurium* [16, 86]. Together, in the absence of a clear cutoff suggested by the tool itself and without TIS data, these strategies should prove to be efficient means to identify novel sORFs. Many putative sORFs were predicted by DeepRibo with better scores than the 18/33 validated sORFs (data not shown), including four novel sORFs with higher ranks than all western blot verified sORFs found by the original study [20]. This suggests that casting a wide net is preferable if additional datasets are available to aid prioritization. While including TIS data might also narrow down a list to higher-confidence candidates, many predicted by DeepRibo were not identified by TIS profiling in the original study [20]. Since antibiotics used for TIS profiling can have different efficiencies on different ORFs [29], this points to the utility of including predictions based on `normal' Ribo-seq data, for example by DeepRibo, REPARATION_blast or smORFer, along with ORF prediction based on start codon signals.

We inspected Ribo-seq coverage for some of the sORFs missed by DeepRibo. The novel sORF *ytgA* (16 aa) was predicted as an N-terminally extended version (Figure 5A). In comparison, validated *yqhJ* (19 aa) was also both labeled as translated and detected by DeepRibo (Figure 5B). However, this candidate has the lowest DeepRibo score (-4.078) and was ranked 5352nd out of all novel sORF candidates, despite having significant Ribo-seq coverage and a strongly enriched TIS peak.

A major challenge in predicting novel ORFs is assigning the correct start codon [44]. smORFer was specifically designed to combine Ribo-seq and TIS libraries together in order to find a set of promising sORF candidates in prokaryotes. The general idea would be to first predict a list of sORFs based on the available Ribo-seq library and then further filter this list using the TIS library to determine the correct start codons. As the coverage of the Ribo-seq library from the original study [20] is low, smORFer was unable to detect any of the novel sORFs using the Ribo-seq library alone. As the TIS library would then be used to further filter the resulting Ribo-seq predictions, smORFer would be unable to detect any of the verified novel sORFs. In contrast to smORFer, DeepRibo trains a cutoff based on the input data, which allows it to adapt to the low coverage of the Ribo-seq library. For smORFer to work well, both Ribo-seq and TIS libraries should be of similar quality or the cutoffs manually adjusted, which currently requires a change in the smORFer scripts (as described in the GitHub documentation). Nevertheless, as smORFer is modular, it allowed us to run the TIS analysis independently. This returned a list of start codons with their respective read counts, based on the TIS library. To compare these start codons to the list of verified novel sORFs, we chose for each start codon the next in-frame stop codon. This enabled us to detect 28 of the 31 novel sORFs. For the missing three sORFs, one was of too low read coverage and the other two were missed by one codon. Using the approach of filtering the start codons, we ended up with a list of 12 381 candidates, which is far too many for manual inspection. As shown in Table 6, the predictions of smORFer (577 being the lowest rank) behave in a similar fashion than the DeepRibo predictions, in a sense that there are far more interesting novel sORF targets to pick first before looking at the verified novel sORFs. As it is unlikely that all of these 12 381 novel sORF candidates are actively translated, a further filtering step would be required. However, this result shows how powerful TIS data can be to determine the correct start codons for a list of candidates. Using the TIS data as suggested in smORFer to filter a strong list of Ribo-seq sORF candidates with conflicting start codons for the predicted stop codons could yield a small list of promising novel sORFs that can be experimentally verified.

The above observations suggest that even bacterial prediction tools require further optimization in the context of novel sORF detection or can be prone to missing true candidates due to expression cutoffs. This was visible for the fixed read count cutoffs of smORFer, which caused it to miss all novel sORFs when considering the Ribo-seq library alone. However, many additional novel sORFs not reported in [20] were detected by DeepRibo and smORFer with a relatively high predictive score or read count, respectively. While some of these might be false positives that can be excluded based on TIS data, others could be candidates for experimental verification. Nonetheless, the ranking system of DeepRibo and the observation of the rank-distribution of verified novel candidates shows that a robust cutoff could improve the usability of DeepRibo. Additionally, the results of smORFer show the power of TIS data to further filter a list of Ribo-seq detected candidates. In this particular case, a combination of the DeepRibo Ribo-seq predictions and the smORFer TIS predictions would likely result in a solid list of sORF candidates. smORFer alone should be sufficient when using a Ribo-seq library with higher read coverage. While prediction tools can always be improved in terms of their specificity, we suggest casting a wide net of predictions based on the availability of additional datasets to validate or screen for interesting candidates. Finally, we recommend inspecting a short list of high-confidence candidates in a genome browser for Ribo-seq coverage patterns and genomic context information that might be missed by current computational approaches as a robust way to identify those for orthogonal validation and future functional characterization.

### Secondary measures

**Figure 7:**
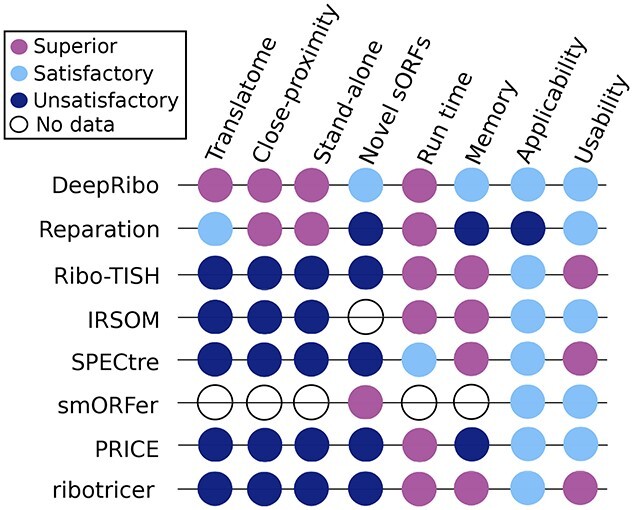
**Summary of tool performance and secondary measures.** Shown are the scored tool prediction performances (AUC) on the whole *translatome* and the subset of close-proximity (200nt) ORFs. The performance on novel *E. coli* sORFs novel ORF is also scored. The last four columns summarize the evaluated quality measures: here run time, memory usage, applicability for experimental design (Applicability) and user-friendliness (Usability) are scored. The systematic approach used to define the performance (Superior, Satisfactory, Unsatisfactory) for the evaluation is described in detail in the supplementary material.

Besides predictive power, other practical considerations can influence the choice of the best tool for ORF detection. We therefore also investigated quantitative (runtime and peak memory usage) and qualitative (usability, applicability) [87] secondary measures for each tool (Figure 7).

Runtime and peak memory usage of the tools were investigated in a single and multi-threading scenario. Runtime and memory were analyzed using the self-generated *E. coli* benchmark set. The size of the associated Ribo-seq *BAM* file is 159 MB (7 457 594 reads) and the RNA-seq *BAM* file 197 MB (9 660 815 reads). The annotation file used includes 4379 annotated coding features. This analysis was run on a cloud instance using 28 VCPUs of an AMD EPYC (with IBPB) processor and 64 GB of RAM, using the taskset utility for all tools.

The best runtimes using only one CPU core were achieved by IRSOM and ribotricer, which completed analysis of the dataset in under 3 min, followed by Ribo-TISH (9 min), PRICE and DeepRibo (approx. 35 min) and REPARATION_blast (>2 h) (Table 7). DeepRibo ignored the maximum number of threads assigned via command-line attribute if the maximum number of cores was not restricted by the operating system, using the taskset command. This behaviour was reproduced on another cloud instance with a different hardware setup. SPECtre had an average runtime compared with the other tools. We did not observe a difference in runtime when providing multiple cores when using the default settings of SPECtre. As smORFer is made up of several modules, we checked the runtime of all modules individually and summed them together. For smORFer, the runtime and memory usage is highly dependant on the modules and the maximum ORF length used. Using smORFer as intended in the documentation requires another separate analysis step that involves manual work and is thus hard to time. When using larger maximum ORF lengths (about 3000nt), the runs failed after several days due to memory overflow (especially when using large alignment files). As smORFer was designed for the detection of sORFs, it is usable in a reasonable amount of time for its intended purpose.

**Table 7:** **Runtime and peak memory consumption.** Runtime and peak memory consumption for each tool running on a virtual machine with either 1 or 10 CPU cores and processing one library of the self-generated *E. coli* dataset. *The smORFer runtime and memory does not include the resources required to manually create a calibrated bam file

	Time [s]		Memory [MB]	
Tools / threads	1	10	1	10
REPARATION_blast	8528	1332	6412	6796
Ribo-TISH	482	62	137	137
DeepRibo	2145	1079	3921	3901
IRSOM	87	—	853	—
SPECtre	3871	3894	1535	1534
smORFer*	12 934	—	11 995	—
PRICE	1851	590	8604	8657
ribotricer	152	—	653	—

On a single core, Ribo-TISH had the lowest peak memory consumption (119 MB), followed by ribotricer (653 MB), IRSOM (853 MB), SPECtre (1535 MB), DeepRibo (3921 MB), REPARATION_blast (6412 MB), and PRICE (8604 MB). smORFer required 11 995 MB, but can go up to our available 64GB if using a higher ORF length cutoff and bigger alignment files.

Applicability of a tool can also contribute to its suitability for a specific task. Ribo-TISH is the only tool out of the eight tested that supports the input of replicates. REPARATION_blast and PRICE, on the other hand, do not produce a deterministic output, meaning that the results of the tool with identical inputs are different between calls. Only PRICE and smORFer use standard output formats (*BED*), whereas DeepRibo can create standard output files (*BEDGRAPH*) via an included postprocessing script. The output of the other tools has to be parsed or converted for downstream analysis by the user (i.e. inspection in a genome browser). Only PRICE uses some unit testing to ascertain the correctness of functions and the reliability of results. Nevertheless, the results of nearly all tools were consistent over different species and annotations. For SPECtre and PRICE, the results were inconsistent and for smORFer, we did not obtain results for all organisms. We scored the applicability of the tools as detailed in the supplemental material (Subsection E.6, Applicability).

Usability determines how user friendly a tool is. We scored the usability of each tool as detailed in the supplemental material (Subsection D.7, Usability). The eight benchmarked tools were stably available from software hosting platforms. Only Ribo-TISH, REPARATION_blast, and ribotricer could be installed with dependencies via a package management system. With the exception of Ribo-TISH, ribotricer, and PRICE, all tools have had a sample dataset available for testing. DeepRibo, Ribo-TISH, ribotricer, and PRICE featured change-logs. They also featured, like SPECtre, a versioning scheme—a key criterion for reproducibility. The documentation of the tools had varying levels of detail and completeness, but all had documented tool dependencies. However, the command line parameters of IRSOM were not documented, DeepRibo was missing documentation concerning its required input, and the output documentation of IRSOM as well as REPARATION was either missing or difficult to find. The published version of SPECtre accepted only Ensembl-formatted *GTF* annotation input, which makes it necessary for many users to specifically preprocess their annotation. All tools were open source, including REPARATION in the REPARATION_blast variant.

## 4 Conclusions

With RiboReport, we aimed to identify the best available tools for Ribo-seq based ORF detection in bacteria using a set of trusted ORFs that we generated using datasets from diverse species. Astoundingly, out of the 13 tools found in literature, only three (DeepRibo, REPARATION_blast, smORFer) were compatible with bacterial annotations and genomes (Table 1), whereas eukaryotic tools like Ribo-TISH, PRICE, SPECtre and ribotricer required features that are not available in NCBI annotations for bacteria, but are often provided in old *GTF* format files from the Ensembl Bacteria FTP server. Adapting the annotation of bacteria to use these features made it possible to run most of the eukaryotic tools with varying levels of success. In addition, the coding potential detection tool IRSOM, which uses only transcriptome data, was added to investigate the performance gain achieved by using Ribo-seq data together with specialized ORF detection tools. While the predictive performance of DeepRibo and REPARATION_blast was superior to the other tools, their runtime and peak memory consumption were substantially higher than for IRSOM, SPECtre, Ribo-TISH and ribotricer. smORFer showed an equally promising predictive performance for sORFs in general and for novel sORFs. However, for the other three datasets, we were not able to calibrate alignment files using candidates with longer ORF lengths (around 3000 nt) without running into memory problems. Before this study was conducted, ribotricer was the only tool available from a package manager. To integrate the tools into our pipeline, we have created either conda packages or docker containers for each of the working tools.

DeepRibo and REPARATION_blast showed a superior predictive performance over SPECtre, Ribo-TISH, ribotricer, PRICE and IRSOM for all organisms and all annotated ORF sets (*translatome*, *sORFs*, *close-proximity genes* and *stand-alone genes*). A set of recently identified and validated sORFs outside of the *E. coli* annotation [20] was used to test novel sORF detection. These sORFs were poorly detected by all tools, with the exception of DeepRibo and smORFer. DeepRibo predicted 17 of the 31 novel sORFs, but most of these predictions did not have a high rank (Table 6). One advantage of DeepRibo is that it learns an RPKM and coverage cutoff based on the input data. When analyzing datasets with low Ribo-seq coverage, this might give it an edge over tools with fixed cutoffs like smORFer. Moreover, DeepRibo provides a neural network that is designed for bacteria and trained on several bacterial datasets. Its pretrained model gives it a certain independence from dataset quality, which allows DeepRibo to work consistently well over several datasets. Tools that retrain their model for each dataset, like REPARATION_blast, are more dependant on the quality of the data. smORFer was initially unable to predict any of the novel sORFs because of the low coverage of the Ribo-seq library used, but when also using the results of the TIS analysis, it showed promising results. Out of the 31 verified novel sORFs, 28 could be detected using the TIS data. While many of these show low read counts, this nevertheless supports the benefit of combining both Ribo-seq and TIS data, as it helps to detect the correct start codon. The detection of the exact ORF boundaries is one of the main problems of tools that are using only classical Ribo-seq libraries [44]. This is one of the reasons we chose a 70% overlap cutoff for predictions, rather than testing for exact matches.

The high sensitivity of DeepRibo appears to come at a cost of a high false positive rate. While a score is generated by the tool to provide a way to sort for higher confidence candidates, a robust cutoff to allow investigation of strong candidates only is not offered. While DeepRibo does a very good job at detecting the correct boundaries based on Ribo-seq coverage alone, it could still benefit from TIS data, especially to reduce the number of false positive predictions. DeepRibo scored distributes shows that it can detect translated ORFs robustly; however, potential novel ORFs are scored very low and are therefore not easy to find (Supplemental Figure S6). However, testing of the top 100 novel sORFs might be a strategy to identify candidate sORFs when no TIS data are available. Some of these false positives might result from highly structured noncoding RNAs, which escape RNase digestion or associate with ribosomes [88]. Further optimization of ORF prediction tools to detect artefacts such as this should be considered in the future.

Most eukaryotic tools like PRICE or ribotricer use very strict expression or coverage cutoffs to filter the final list of candidate ORFs, which is required for eukaryotic data due to the number of predictions. However, these cutoffs are likely too stringent for bacterial data. ribotricer has an automatic cutoff detection, which shows the contrast of these cutoffs. While ribotricer uses a default phase-score cutoff value of 0.428, the automatically detected cutoff for our *E. coli* dataset is just 0.088. This might be one explanation for the lower performance of the eukaryotic tools as it shows how cutoff values can differ between eukaryotes and prokaryotes.

For the tools that we could not test, there was no mention of their taxonomic scope or if they are applicable beyond the scope of what they had been designed and tested on. Ribo-TISH, while unsatisfactory in terms of predictive power, was also clearly not designed with bacterial data in mind. However, it was the only tool that supports replicates as input. Furthermore, Ribo-TISH and smORFer are the only tools that support TIS data. As TIS profiling is now established in bacteria and archaea [19, 20, 89], we expect this to be an essential capability of future tools. Looking to the future, we hope that support for TIS data, replicates, and nonstandard organisms is considered in new tools or improved versions of the current tools, as smORFer clearly shows the benefits of start codon detection based on TIS data.

**Key Points**

- Generated an ORF dataset for benchmarking ORF prediction tools using Ribo-seq data in bacteria.
- Created a benchmarking pipeline that can be extended with additional tools for future testing.
- DeepRibo is the first choice for bacterial ORF prediction tasks using Ribo-seq data alone.
- Tool performance was comparable between ORFs translated from ORFs in close proximity to other genes versus stand-alone ORFs.
- Identification of relatively high confidence novel sORFs by DeepRibo is likely possible by selecting the top 100 novel candidates sorted by score for further manual inspection.
- A significant number of sORFs recently discovered using TIS profiling are not detected by tools despite sufficient Ribo-seq signal.
- smORFer shows the strong potential of using TIS data to determine correct start codons for candidate ORFs.
- Tools should embrace the use of replicates, TIS profiling data, and also include improved software packaging, usability, and documentation.

## 5 Author contributions statement

F.E., R.G., S.L.S., T.M. and R.B. designed the study; S.L.S. performed the experiments; F.E., S.L.S., R.G. and T.M. screened databases for bacterial Ribo-seq datasets; S.L.S. performed manual labeling of the translated regions; F.E. retrieved and processed the proteomics data; O.S.A. computed the ORFs in close proximity to other ORFs; R.G. performed high throughput sequencing analysis, tool testing and ORF predictions; T.M. performed the benchmark analysis and created the benchmark plots; R.B. and C.M.S. supervised the project and provided funding. All authors jointly wrote the manuscript.

## Supplementary Material

supplement_bbab549

## Notes

**Rick Gelhausen** is a PhD student in the Bioinformatics group, Department of Computer Science of the Faculty of Engineering, at the Albert-Ludwigs-University of Freiburg, Germany. His current research focuses on ribosome profiling analysis with a special interest in the detection of novel small open reading frames.

**Teresa Müller** is a PhD student in the Bioinformatics group, Department of Computer Science of the Faculty of Engineering, at the Albert-Ludwigs-University of Freiburg, Germany. Her research focuses on RNA-RNA interactions and sequence-structure alignments with a special interest in benchmarking.

**Sarah Lauren Svensson** is postdoctoral researcher in the Department of Molecular Infection Biology II at the University of Würzburg in Germany. Her research interests include the application of deep sequencing approaches to study stress responses/virulence, functional genomics, and transcriptomes/translatomes of bacterial pathogens, as well as the functional characterization of small regulatory RNAs and small proteins involved in pathogenesis.

**Omer Salem Alknbashi** is an assistant professor in the department of Information and Computer Science (ICS) at King Fahd University of Petroleum & Minerals (KFUPM), Saudi Arabia. He has a PhD in Bioinformatics from the University of Freiburg, 2017. His area of research is Computational Characterisation of Genomic CRISPR-Cas Systems in Archaea and Bacteria. In addition, he is interested in biological analysis data (metagenomics, HTS data, evolution) and machine learning.

**Cynthia Mira Sharma** is Chair of Molecular Infection Biology II at the Julius-Maximilians University of Würzburg. Her research focuses on developing and adapting deep sequencing approaches to study small regulatory RNAs, RNA binding proteins, CRISPR/Cas systems, small proteins, and virulence of the pathogenic Epsilonproteobacteria *Campylobacter jejuni* and *Helicobacter pylori*.

**Florian Eggenhofer** is postdoctoral researcher in the Bioinformatics group, Department of Computer Science of the Faculty of Engineering, at the Albert-Ludwigs-University of Freiburg, Germany. His research interests includes RNA bioinformatics, homology searches, high throughput sequencing, and detection and analysis of open reading frames.

**Rolf Backofen** is head of the Bioinformatics group, Department of Computer Science of the Faculty of Engineering, at the Albert-Ludwigs-University Freiburg, Germany. His main research interests include molecular interactions of RNAs and proteins, the detection of RNA sequence/structure motifs, and the clustering of molecular sequences such as like CRISPR/Cas.
