# Supplementary material for: RiboReport - benchmarking tools for ribosome profiling-based identification of open reading frames in bacteria
Source: Brief Bioinform. 2022 Jan 17;23(2):bbab549. doi: 10.1093/bib/bbab549 (PMC8921622; doi:10.1093/bib/bbab549)
Supplement: supplement_bbab549 [file supplement_bbab549.pdf]

RiboReport - Supplemental Material

Rick Gelhausen[1], Teresa Müller[1], Sarah L. Svensson[3], Omer S. Alkhnbashi[1], Cynthia M. Sharma[3], Florian Eggenhofer[1], Rolf Backofen[1,2]

[1] Bioinformatics Group, Department of Computer Science University of Freiburg, Georges-Köhler-Allee, 79110 Freiburg, Germany
[2] Centre for Biological Signalling Studies (BIOSS), University of Freiburg, 79110 Freiburg, Germany
[3] Department of Molecular Infection Biology II, Institute for Molecular Infection Biology, University of Würzburg, Josef-Schneider-Str. 2 / D15, 97080 Würzburg, Germany

Table of Contents

A Introduction . . . 2
B Validation of labeling method . . . 2
C Supplementary Result Figures . . . 3
D Supplementary Result Tables . . . 9
D.1 Translatome . . . 9
D.2 Close-proximity genes . . . 14
D.3 Stand-alone genes . . . 18
D.4 Small Open Reading Frames . . . 22
D.5 Novel sORF detection . . . 25
E Evaluation of key results . . . 25
E.1 Predictive power for the *translatome* set . . . 25
E.2 Predictive power inside and outside of operons . . . 25
E.3 Prediction of novel sORFs . . . 26
E.4 Runtime . . . 26
E.5 Memory . . . 26
E.6 Applicability . . . 27
E.7 Usability . . . 27
F Adapter sequences used for trimming . . . 29
F.1 *E. coli* . . . 29
F.2 *L. monocytogenes* . . . 29
F.3 *P. aeruginosa* . . . 29
F.4 *S.* Typhimurium . . . 29

A Introduction

This document contains supplemental material for *RiboReport - Benchmarking tools for ribosome profiling-based identification of open reading frames in bacteria.*

B Validation of labeling method

We validated our labeling approach by comparison to available published MS datasets (proteomics) for the same strains grown under similar conditions. Comparing the translated ORFs found by our labeling method to those detected in the retrieved proteomics data for each organism showed that the majority of genes labeled as translated based on Ribo-seq were also detected by MS (Supplemental Figure S1)(on average 83.81% across all four organisms), thereby validating our labeling procedure.

To further showcase the overlap of human labeling in the genome browser versus MS to generate a robust benchmark set, we selected a set of conserved

and highly translated ORFs that could be compared in all four organisms: a long ribosomal protein island that is highly conserved in bacteria (genes *rpmJ* to *secY*) and also features several sORFs with less than 50 aa. The genome browser tracks show that for *E. coli*, *P. aeruginosa*, and *L. monocytogenes*, both MS and labeling detected all 22 of these ORFs and that the entire island showed strong Ribo-seq coverage (Supplemental Figures S2A - S2C). In contrast, while all 22 ORFs were labeled as translated in *S.* Typhimurium only five were detected by MS, consistent with the generally lower sensitivity of the *S.* Typhimurium MS dataset (Figure S1).

As an additional check of our labeling procedure, we also labeled a selection of tRNAs and annotated non-coding RNAs based on our *de novo E. coli* dataset (data not shown). None of the tRNAs examined (86) were called as translated based on comparison of Ribo-seq and RNA-seq coverage. Several well-characterized ncRNAs were also labeled as not translated, such as the regulatory small RNAs MicA (Supplemental Figure S3A), Spot 42, SdsR, and GlmY, as well as the housekeeping RNA components of the signal recognition particle (SRP RNA) and RNase P (RnpB) (data not shown). However, some known non-coding RNAs were labeled as translated, such as CsrC (Supplemental Figure S3B) and tmRNA (data not shown). Some of these false positives could be due to resistance to RNase digestion or association with ribosomes (tmRNA). However, some, such as the annotated non-coding RNA RyeG, were recently shown to encode translated sORFs (Supplemental Figure S3C, *yodE*, 48 aa) [1]. We also inspected an ORF that was detected by MS, but not labeled as translated, *katE*. This ORF was not labeled as translated because of low coverage (generally below 10 relative reads) (Supplemental Figure S3D). Its detection by MS suggests either the conditions for Ribo-seq and MS were slightly different, or that KatE is a relatively stable protein that is translated at an earlier growth phase from an mRNA that is not highly expressed during exponential growth. Nonetheless, while these examples (Supplemental Figure S3B and S3D) highlight some limitations of our labeling approach, overall the overlap of labels and MS, together with accurate labeling of known non-coding RNAs and sORFs, demonstrates the validity of our approach.

C Supplementary Result Figures

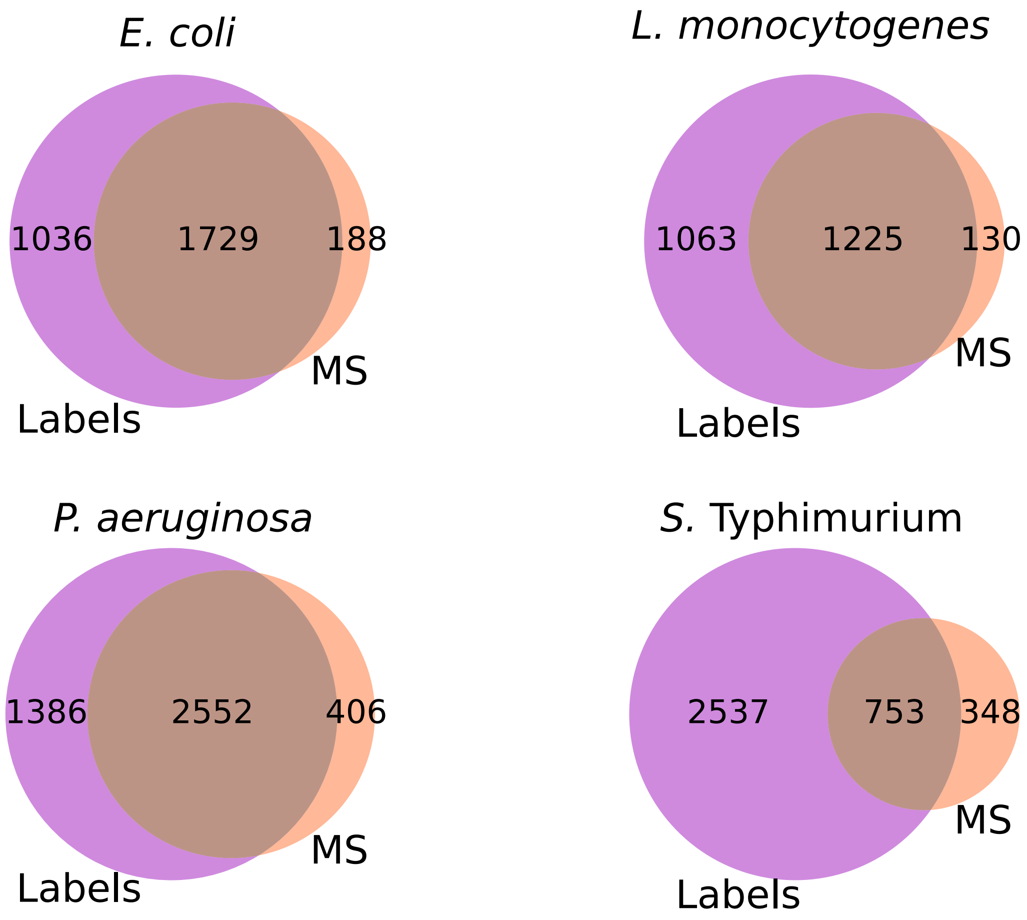

Fig. S1: Comparison between ORFs labeled as translated with Ribo-seq data and ORFs detected with proteomics. The intersection between annotated ORFs labeled as translated based on Ribo-seq data (Labels) and those detected by mass spectrometry (MS) for the four benchmark datasets. The majority of the genes detected by MS are also present in the labeled benchmark dataset. The percent of MS ORFs that were also found by our labeling method for each organism are as follows: *E. coli*, 90%; *L. monocytogenes*, 90%; *P. aeruginosa*, 86%; *S.* Typhimurium, 68%; and on average 83.8%. The large overlap validates the manual labeling strategy we employed to create the benchmark datasets.

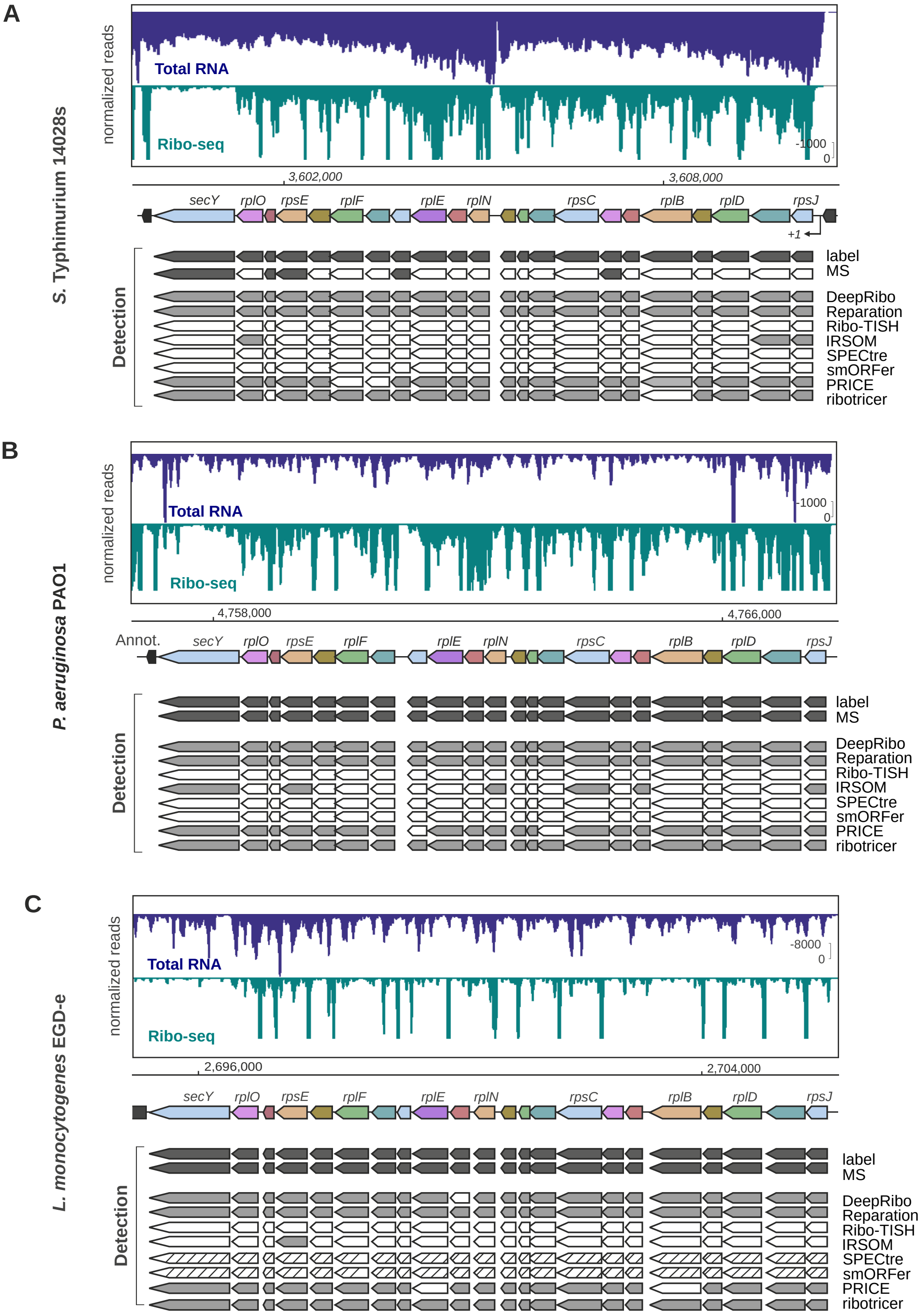

Fig. S2: Comparison of manual Ribo-seq labels and proteomics (MS) for conserved ribosomal protein ORFs from *S.* Typhimurium (A), *P. aeruginosa* (B), and *L. monocytogenes* (C). Related to Main Figure 3. The conserved region between *rpmJ* and *secY* encodes several highly expressed ORFs of diverse length. Corresponding genes between the organisms are labeled with the same colour in the annotation. Genes that are detected in the publicly available MS dataset and by manual curation of the Ribo-seq data (label) are dark grey. Detection by the indicated tools at a 70% overlap threshold based on Ribo-seq data (or RNA-seq, `IRSOM`) is indicated in light grey. Those that are not detected are white. Transcriptional start sites, if available, are indicated with a bent arrow (+1). Hatched arrows indicate that predictions could not be generated for the genome/dataset.

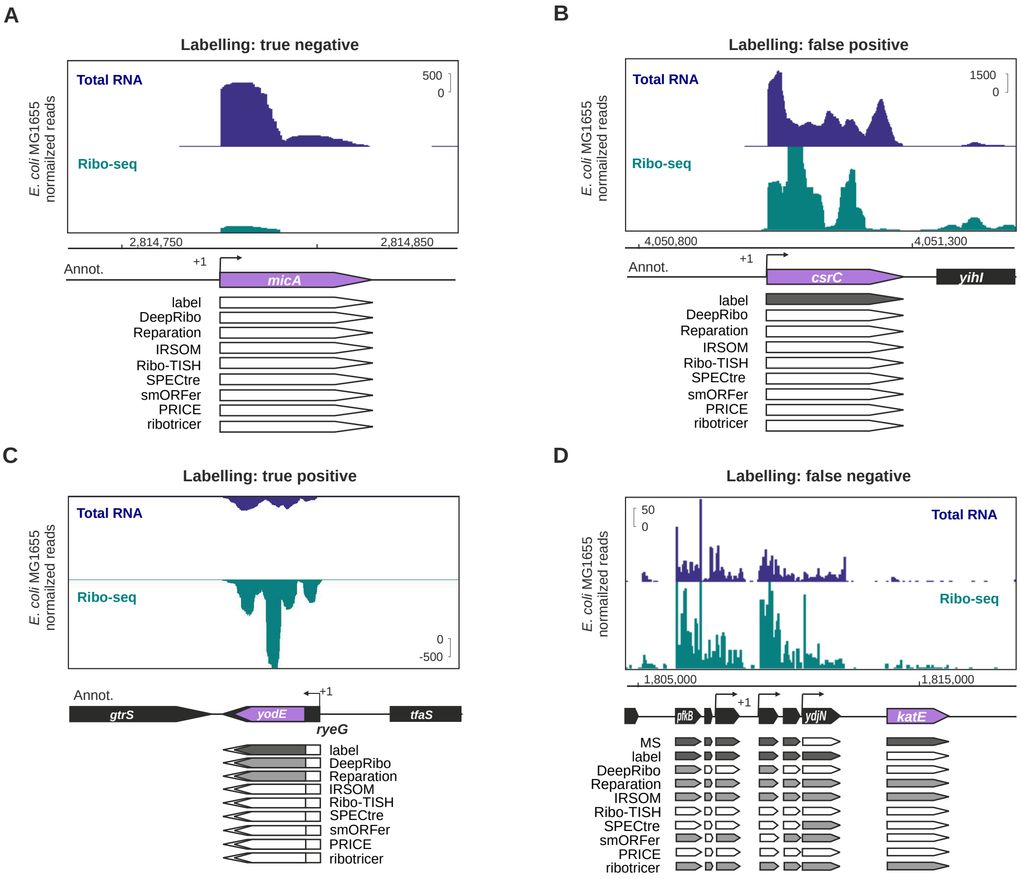

Fig. S3: Assessing the quality/accuracy of manual curation for well-characterized and/or validated *E. coli* genes. An example of a true negative, false positive, true positive, and false negative manual curation from the *E. coli* dataset were selected. **(A)** The non-coding base-pairing regulatory RNA MicA was correctly labeled as not translated based on Ribo-seq coverage and is a true-negative. **(B)** The non-coding RNA CsrC shows coverage in the Ribo-seq library and was false-positively labeled as translated. **(C)** The newly validated sORF true-positive *yodE* (48 aa) [1], encoded on the annotated RyeG sRNA, was labeled as translated based on Ribo-seq. **(D)** The *katE* ORF was not labeled as translated and shows low coverage in the RNA-seq and Ribo-seq libraries, but has been detected by MS under similar growth conditions. Tool predictions and MS data for annotated coding genes are also included for comparison. Detection (grey arrows) or no detection (white arrows) was determined at a 70% overlap threshold.

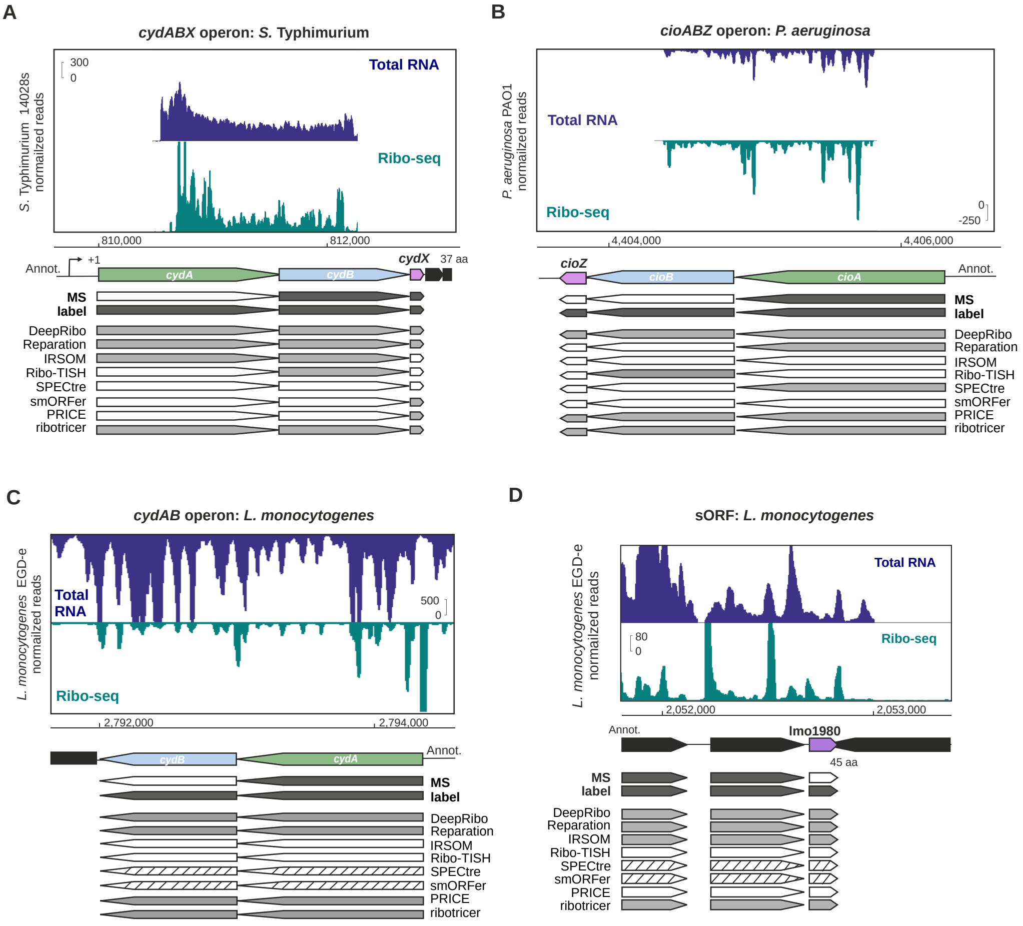

Fig. S4: Comparison of detection of corresponding ORFs from the translatome and small ORFs sets by the four tested tools. (A) *S.* Typhimurium *cydABX*. **(B)** *P. aeruginosa cioABZ* operon. **(C)** *L. monocytogenes cydAB* operon. **(D)** The validated *L. monocytogenes* sORF lmo1980 (45 aa). The ORFs annotated in syntenic *cydAB*/*cioAB* regions, encoding terminal oxidase complexes, were inspected for MS detection, detection based on Ribo-seq by either manual labeling or the computational tools. Corresponding genes between the organisms are labeled in the same colour in the annotation track. Genes encoding associated small protein components of the complexes (pink arrow) are annotated so far only in *P. aeruginosa* (*cioZ*) and have so far not been detected in the *L. monocytogenes cydAB* operon [2]. Detection (grey arrows) or no detection (white arrows) was determined at a 70% overlap threshold. Hatched genes indicate that predictions could not be generated for the genome/dataset. Spaces between *cydA* and *cydB* are as follows: *S.* Typhimurium: 15 nt, *P. aeruginosa*: 3 nt, and *L. monocytogenes*, 14 nt overlap. For all panels, detection (grey arrows) or no detection (white arrows) was determined at a 70% overlap threshold. Hatched arrows indicate that predictions could not be generated for the genome/dataset.

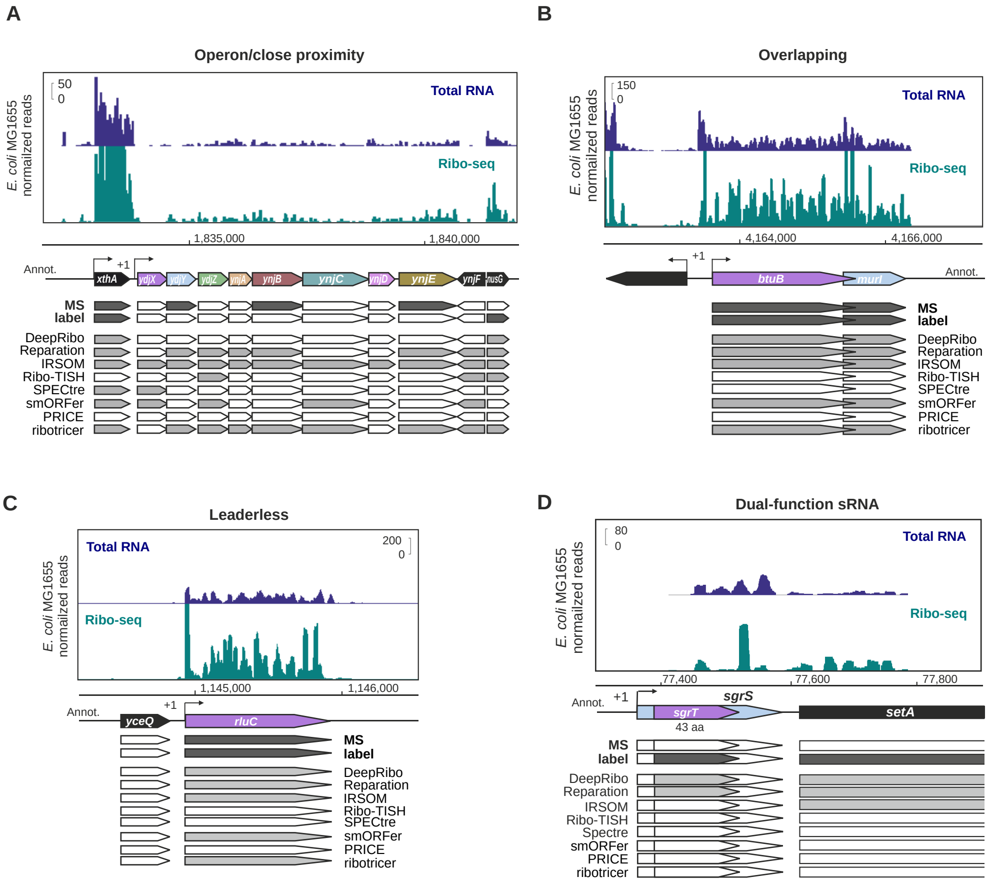

Fig. S5: Detection of examples of ORFs in bacteria-specific contexts in *E. coli.* set of eight lowly-transcribed polycistronic ORFs (*ydjX*-*ynjE*) was not labeled as translated based on Ribo-seq because of low overall coverage, but protein products from three of these ORFs were detected by MS under similar growth conditions. **(B)** The overlapping *btuB*-*murI* operon. Both ORFs were detected in the MS dataset, labeled as translated based on Ribo-seq, and were also identified by `DeepRibo`, `REPARATION blast`, ribotricer, smORFer, and `IRSOM`. **(C)** The leaderless ORF *rluC*, which was detected by MS and labeled based on Ribo-seq, was also detected by the prediction tools `DeepRibo`, `REPARATION_blast`, `smORFer`, `ribotricer`, and `IRSOM`, even without a Shine-Dalgarno sequence. **(D)** The dual function RNA SgrS acts as both a base-pairing repressor, and also encodes the small protein SgrT (43 aa [3]. SgrT was not detected by MS under these conditions but was labeled as translated based on Ribo-seq coverage and was detected by `DeepRibo` and `REPARATION_blast`. For all panels, detection (grey arrows) or no detection (white arrows) was determined at a 70% overlap threshold.

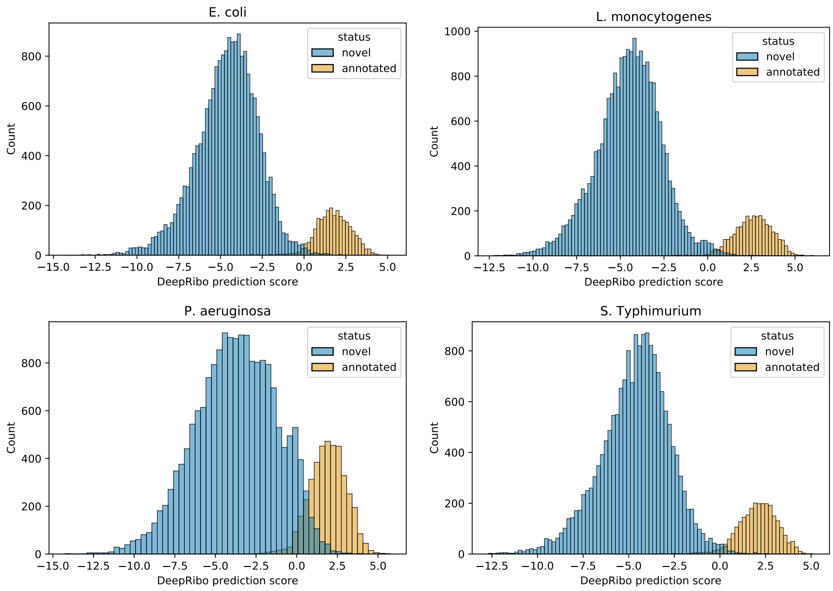

Fig. S6: DeepRibo prediction score distribution. The predictions are split into annotated (orange) and novel (blue) ORF predictions. Novel predictions are those having a score of '-1' for the 'dist' parameter, as described in the DeepRibo documentation. For *E. coli* the prediction score of the last predicted annotated ORF is -12.14 and the number of novel predictions after this ORF is 42,879. For *L. monocytogenes* the prediction score of the last predicted annotated ORF is -11.13 and the number of novel predictions after this ORF is 45,282. For *P. aeruginosa* the prediction score of the last predicted annotated ORF is -12.01 and the number of novel predictions after this ORF is 37,470. For *S.* Typhimurium the prediction score of the last predicted annotated ORF is -14.8 and the number of novel predictions after this ORF is 36,272.

D Supplementary Result Tables

The following supplemental result tables are also available as Google Spreadsheets for exporting and further analysis.

D.1 Translatome

Table S1: Statistical evaluation of tool performance for *translatome* ORFs for the *E. coli* benchmark dataset using overlap thresholds of 0.01, 0.7, or 0.9. The overlap threshold is the percent of the ORF length that must overlap with the prediction. The overlap threshold needs to be fulfilled both for prediction with labeled ORF and vice versa. The true positives (TP), true negatives (TN), false positives (FP), and false negatives (FN) are based on the numbers of ORFs or predictions given the current overlap threshold. The sensitivity or TP rate (TPR), specificity or TN rate (TNR), FN rate (FNR), precision or positive predictive value (PPV), false discovery rate (FDR), F1 measure, and the accuracy are calculated based on the given TP, TN, FP, and FN values. Since more than one prediction meeting the specified overlap threshold can overlap with one ORF, the number of additional overlapping predictions are reported in the suboptimal TP (sTP) and suboptimal FP (sFP) columns. The number of prediction which do not overlap, given the overlap cutoff, with any annotated gene are displayed int the column no gene found (no_gene)

TP	TN	FP	FN	TPR	TNR	FNR	PPV	FDR	F1	accuracy	sTP	sFP	no_gene	tool
E. coli **and using overlap of 0.01**														
2704	644	841	59	0.98	0.43	0.02	0.76	0.24	0.86	0.79	58	8	149	`REPARATION_blast`
1751	1216	269	1012	0.63	0.82	0.37	0.87	0.13	0.73	0.70	2571	157	81	`Ribo-TISH`
2670	1205	280	93	0.97	0.81	0.03	0.91	0.09	0.93	0.91	16315	342	2452	`DeepRibo`
1470	755	730	1293	0.53	0.51	0.47	0.67	0.33	0.59	0.52	55	33	191	`IRSOM`
1095	800	685	1668	0.40	0.54	0.60	0.62	0.38	0.48	0.45	2	2	0	`SPECtre`
2036	1394	91	727	0.74	0.94	0.26	0.96	0.04	0.83	0.81	5263	28	657	`PRICE`
2544	507	978	219	0.92	0.34	0.08	0.72	0.28	0.81	0.72	24	8	414	`ribotricer`
2366	568	917	397	0.86	0.38	0.14	0.72	0.28	0.78	0.69	2689	395	1403	`smORFer`
E. coli **and using overlap of 0.7**														
2693	709	776	70	0.97	0.48	0.03	0.78	0.22	0.86	0.80	1	0	272	`REPARATION_blast`
84	1416	69	2679	0.03	0.95	0.97	0.55	0.45	0.06	0.35	59	26	4589	`Ribo-TISH`
2305	1437	48	458	0.83	0.97	0.17	0.98	0.02	0.90	0.88	17	1	19664	`DeepRibo`
1425	782	703	1338	0.52	0.53	0.48	0.67	0.33	0.58	0.52	6	7	320	`IRSOM`
1094	802	683	1669	0.40	0.54	0.60	0.62	0.38	0.48	0.45	0	0	0	`SPECtre`
326	1451	34	2437	0.12	0.98	0.88	0.91	0.09	0.21	0.42	1	0	7711	`PRICE`
2542	510	975	221	0.92	0.34	0.08	0.72	0.28	0.81	0.72	3	1	432	`ribotricer`
1138	1080	405	1625	0.41	0.73	0.59	0.74	0.26	0.53	0.52	3	2	6210	`smORFer`
E. coli **and using overlap of 0.9**														
2650	773	712	113	0.96	0.52	0.04	0.79	0.21	0.87	0.81	0	0	380	`REPARATION_blast`
51	1444	41	2712	0.02	0.97	0.98	0.55	0.45	0.04	0.35	9	4	4722	`Ribo-TISH`
2246	1440	45	517	0.81	0.97	0.19	0.98	0.02	0.89	0.87	3	0	19741	`DeepRibo`
1425	782	703	1338	0.52	0.53	0.48	0.67	0.33	0.58	0.52	2	2	329	`IRSOM`
1094	802	683	1669	0.40	0.54	0.60	0.62	0.38	0.48	0.45	0	0	0	`SPECtre`
234	1456	29	2529	0.08	0.98	0.92	0.89	0.11	0.15	0.40	0	0	7809	`PRICE`
2542	510	975	221	0.92	0.34	0.08	0.72	0.28	0.81	0.72	0	0	436	`ribotricer`
728	1265	220	2035	0.26	0.85	0.74	0.77	0.23	0.39	0.47	0	0	6810	`smORFer`

Table S2: Statistical evaluation of the *translatome* ORFs for the benchmark dataset *L. monocytogenes* using overlap thresholds of 0.01, 0.7, or 0.9. A detailed column description can be found in main Table S1.

TP	TN	FP	FN	TPR	TNR	FNR	PPV	FDR	F1	accuracy	sTP	sFP	no_gene	tool
L. monocytogenes **and using overlap of 0.01**														
1946	335	244	342	0.85	0.58	0.15	0.89	0.11	0.87	0.80	58	8	47	`REPARATION_blast`
1023	390	189	1265	0.45	0.67	0.55	0.84	0.16	0.58	0.49	695	110	80	`Ribo-TISH`
2284	56	523	4	1.00	0.10	0.00	0.81	0.19	0.90	0.82	15433	2051	4211	`DeepRibo`
985	290	289	1303	0.43	0.50	0.57	0.77	0.23	0.55	0.44	34	21	62	`IRSOM`
-	-	-	-	-	-	-	-	-	-	-	-	-	-	`SPECtre`
2164	227	352	124	0.95	0.39	0.05	0.86	0.14	0.90	0.83	12664	673	3007	`PRICE`
2288	4	575	0	1.00	0.01	0.00	0.80	0.20	0.89	0.80	41	31	238	`ribotricer`
L. monocytogenes **and using overlap of 0.7**														
1866	367	212	422	0.82	0.63	0.18	0.90	0.10	0.85	0.78	0	0	207	`REPARATION_blast`
55	556	23	2233	0.02	0.96	0.98	0.71	0.29	0.05	0.21	32	18	1967	`Ribo-TISH`
2194	215	364	94	0.96	0.37	0.04	0.86	0.14	0.91	0.84	4	1	21864	`DeepRibo`
962	298	281	1326	0.42	0.51	0.58	0.77	0.23	0.54	0.44	6	1	108	`IRSOM`
-	-	-	-	-	-	-	-	-	-	-	-	-	-	`SPECtre`
461	565	14	1827	0.20	0.98	0.80	0.97	0.03	0.33	0.36	0	0	18364	`PRICE`
2288	4	575	0	1.00	0.01	0.00	0.80	0.20	0.89	0.80	7	1	269	`ribotricer`
L. monocytogenes **and using overlap of 0.9**														
1837	374	205	451	0.80	0.65	0.20	0.90	0.10	0.85	0.77	0	0	243	`REPARATION_blast`
36	564	15	2252	0.02	0.97	0.98	0.71	0.29	0.03	0.21	7	2	2035	`Ribo-TISH`
2155	224	355	133	0.94	0.39	0.06	0.86	0.14	0.90	0.83	0	0	21916	`DeepRibo`
962	298	281	1326	0.42	0.51	0.58	0.77	0.23	0.54	0.44	3	1	111	`IRSOM`
-	-	-	-	-	-	-	-	-	-	-	-	-	-	`SPECtre`
448	570	9	1840	0.20	0.98	0.80	0.98	0.02	0.33	0.36	0	0	18382	`PRICE`
2288	4	575	0	1.00	0.01	0.00	0.80	0.20	0.89	0.80	2	0	273	`ribotricer`

Table S3: Statistical evaluation of the *translatome* ORFs for the benchmark dataset *P. aeruginosa* and using overlap threshold of 0.01, 0.7, or 0.9. A detailed column description can be found in main Table S1.

TP	TN	FP	FN	TPR	TNR	FNR	PPV	FDR	F1	accuracy	sTP	sFP	no_gene	tool
P. aeruginosa **and using overlap of 0.01**														
2452	1326	312	1483	0.62	0.81	0.38	0.89	0.11	0.73	0.68	57	6	60	REPARATION_blast
2776	1013	625	1159	0.71	0.62	0.29	0.82	0.18	0.76	0.68	3143	335	111	Ribo-TISH
3869	1014	624	66	0.98	0.62	0.02	0.86	0.14	0.92	0.88	12954	790	3990	DeepRibo
2514	455	1183	1421	0.64	0.28	0.36	0.68	0.32	0.66	0.53	145	117	204	IRSOM
101	1339	299	3834	0.03	0.82	0.97	0.25	0.75	0.05	0.26	0	0	0	SPECtre
3349	1263	375	586	0.85	0.77	0.15	0.90	0.10	0.87	0.83	3278	45	359	PRICE
3738	273	1365	197	0.95	0.17	0.05	0.73	0.27	0.83	0.72	22	10	474	ribotricer
P. aeruginosa **and using overlap of 0.7**														
2271	1350	288	1664	0.58	0.82	0.42	0.89	0.11	0.70	0.65	6	0	304	REPARATION_blast
143	1562	76	3792	0.04	0.95	0.96	0.65	0.35	0.07	0.31	79	36	6647	Ribo-TISH
3689	1378	260	246	0.94	0.84	0.06	0.93	0.07	0.94	0.91	661	45	17408	DeepRibo
2423	504	1134	1512	0.62	0.31	0.38	0.68	0.32	0.65	0.53	11	15	479	IRSOM
101	1339	299	3834	0.03	0.82	0.97	0.25	0.75	0.05	0.26	0	0	0	SPECtre
2121	1447	191	1814	0.54	0.88	0.46	0.92	0.08	0.68	0.64	3	0	5073	PRICE
3738	276	1362	197	0.95	0.17	0.05	0.73	0.27	0.83	0.72	3	0	480	ribotricer
P. aeruginosa **and using overlap of 0.9**														
2097	1365	273	1838	0.53	0.83	0.47	0.88	0.12	0.67	0.62	1	0	498	REPARATION_blast
71	1591	47	3864	0.02	0.97	0.98	0.60	0.40	0.04	0.30	12	5	6846	Ribo-TISH
3571	1406	232	364	0.91	0.86	0.09	0.94	0.06	0.92	0.89	127	15	18118	DeepRibo
2421	506	1132	1514	0.62	0.31	0.38	0.68	0.32	0.65	0.53	5	7	497	IRSOM
101	1339	299	3834	0.03	0.82	0.97	0.25	0.75	0.05	0.26	0	0	0	SPECtre
1543	1518	120	2392	0.39	0.93	0.61	0.93	0.07	0.55	0.55	0	0	5725	PRICE
3738	276	1362	197	0.95	0.17	0.05	0.73	0.27	0.83	0.72	1	0	482	ribotricer

Table S4: Statistical evaluation of the *translatome* for the benchmark dataset *S.* Typhimurium and using overlap threshold of 0.01, 0.7 or 0.9. A detailed column description can be found in Table S1.

TP	TN	FP	FN	TPR	TNR	FNR	PPV	FDR	F1	accuracy	sTP	sFP	no_gene	tool
***S.* Typhimurium** and using overlap of **0.01**														
3056	1076	611	228	0.93	0.64	0.07	0.83	0.17	0.88	0.83	80	29	30	REPARATION_blast
2032	1451	236	1252	0.62	0.86	0.38	0.90	0.10	0.73	0.70	2937	165	37	Ribo-TISH
3088	1462	225	196	0.94	0.87	0.06	0.93	0.07	0.94	0.92	15588	109	1307	DeepRibo
1677	853	834	1607	0.51	0.51	0.49	0.67	0.33	0.58	0.51	80	57	89	IRSOM
142	1303	384	3142	0.04	0.77	0.96	0.27	0.73	0.07	0.29	3	3	0	SPECtre
2210	1644	43	1074	0.67	0.97	0.33	0.98	0.02	0.80	0.78	3703	3	450	PRICE
3116	715	972	168	0.95	0.42	0.05	0.76	0.24	0.85	0.77	52	57	20	ribotricer
***S.* Typhimurium** and using overlap of **0.7**														
3017	1168	519	267	0.92	0.69	0.08	0.85	0.15	0.88	0.84	0	0	169	REPARATION_blast
317	1599	88	2967	0.10	0.95	0.90	0.78	0.22	0.17	0.39	258	42	4674	Ribo-TISH
2521	1657	30	763	0.77	0.98	0.23	0.99	0.01	0.86	0.84	19	1	17642	DeepRibo
1642	897	790	1642	0.50	0.53	0.50	0.68	0.32	0.57	0.51	10	9	185	IRSOM
142	1304	383	3142	0.04	0.77	0.96	0.27	0.73	0.07	0.29	0	0	0	SPECtre
900	1676	11	2384	0.27	0.99	0.73	0.99	0.01	0.43	0.52	0	0	5473	PRICE
3114	732	955	170	0.95	0.43	0.05	0.77	0.23	0.85	0.77	0	0	20	ribotricer
***S.* Typhimurium** and using overlap of **0.9**														
2921	1212	475	363	0.89	0.72	0.11	0.86	0.14	0.87	0.83	0	0	309	REPARATION_blast
218	1626	61	3066	0.07	0.96	0.93	0.78	0.22	0.12	0.37	43	9	5048	Ribo-TISH
2459	1661	26	825	0.75	0.98	0.25	0.99	0.01	0.85	0.83	1	0	17727	DeepRibo
1641	897	790	1643	0.50	0.53	0.50	0.68	0.32	0.57	0.51	3	1	201	IRSOM
142	1304	383	3142	0.04	0.77	0.96	0.27	0.73	0.07	0.29	0	0	0	SPECtre
726	1677	10	2558	0.22	0.99	0.78	0.99	0.01	0.36	0.48	0	0	5648	PRICE
3114	732	955	170	0.95	0.43	0.05	0.77	0.23	0.85	0.77	0	0	20	ribotricer

D.2 Close-proximity genes

Table S5: Statistical evaluation of ORFs located in operons for the benchmark dataset *E. coli* and using overlap threshold of 0.01, 0.7, or 0.9. A detailed column description can be found in main Table S1.

TP	TN	FP	FN	TPR	TNR	FNR	PPV	FDR	F1	accuracy	sTP	sFP	tool
E. coli **and using overlap of 0.01**													
1766	451	564	28	0.98	0.44	0.02	0.76	0.24	0.86	0.79	37	7	REPARATION_blast
1128	840	175	666	0.63	0.83	0.37	0.87	0.13	0.73	0.70	1666	97	Ribo-TISH
1727	849	166	67	0.96	0.84	0.04	0.91	0.09	0.94	0.92	10444	164	DeepRibo
919	514	501	875	0.51	0.51	0.49	0.65	0.35	0.57	0.51	23	16	IRSOM
683	521	494	1111	0.38	0.51	0.62	0.58	0.42	0.46	0.43	2	3	SPECtre
1330	964	51	464	0.74	0.95	0.26	0.96	0.04	0.84	0.82	3554	15	PRICE
1664	344	671	130	0.93	0.34	0.07	0.71	0.29	0.81	0.71	17	7	ribotricer
1568	348	667	226	0.87	0.34	0.13	0.70	0.30	0.78	0.68	1815	285	smorfer
E. coli **and using overlap of 0.7**													
1754	493	522	40	0.98	0.49	0.02	0.77	0.23	0.86	0.80	0	0	REPARATION_blast
54	969	46	1740	0.03	0.95	0.97	0.54	0.46	0.06	0.36	38	12	Ribo-TISH
1490	986	29	304	0.83	0.97	0.17	0.98	0.02	0.90	0.88	8	0	DeepRibo
909	526	489	885	0.51	0.52	0.49	0.65	0.35	0.57	0.51	1	4	IRSOM
681	523	492	1113	0.38	0.52	0.62	0.58	0.42	0.46	0.43	0	0	SPECtre
221	998	17	1573	0.12	0.98	0.88	0.93	0.07	0.22	0.43	0	0	PRICE
1661	348	667	133	0.93	0.34	0.07	0.71	0.29	0.81	0.72	1	1	ribotricer
735	723	292	1059	0.41	0.71	0.59	0.72	0.28	0.52	0.52	1	2	smorfer
E. coli **and using overlap of 0.9**													
1724	534	481	70	0.96	0.53	0.04	0.78	0.22	0.86	0.80	0	0	REPARATION_blast
37	990	25	1757	0.02	0.98	0.98	0.60	0.40	0.04	0.37	7	2	Ribo-TISH
1456	988	27	338	0.81	0.97	0.19	0.98	0.02	0.89	0.87	1	0	DeepRibo
909	526	489	885	0.51	0.52	0.49	0.65	0.35	0.57	0.51	1	1	IRSOM
681	523	492	1113	0.38	0.52	0.62	0.58	0.42	0.46	0.43	0	0	SPECtre
157	1002	13	1637	0.09	0.99	0.91	0.92	0.08	0.16	0.41	0	0	PRICE
1661	348	667	133	0.93	0.34	0.07	0.71	0.29	0.81	0.72	0	0	ribotricer

Table S6: Statistical evaluation of ORFs located in operons for the benchmark dataset *L. monocytogenes* and using overlap threshold of 0.01, 0.7 or 0.9. A detailed column description can be found in Table S1.

TP	TN	FP	FN	TPR	TNR	FNR	PPV	FDR	F1	accuracy	sTP	sFP	tool
L. monocytogenes **and using overlap of 0.01**													
1391	241	191	231	0.86	0.56	0.14	0.88	0.12	0.87	0.79	45	7	`REPARATION_blast`
723	291	141	899	0.45	0.67	0.55	0.84	0.16	0.58	0.49	472	82	`Ribo-TISH`
1619	45	387	3	1.00	0.10	0.00	0.81	0.19	0.89	0.81	10842	1537	`DeepRibo`
670	213	219	952	0.41	0.49	0.59	0.75	0.25	0.53	0.43	15	15	`IRSOM`
-	-	-	-	-	-	-	-	-	-	-	-	-	`SPECtre`
1525	176	256	97	0.94	0.41	0.06	0.86	0.14	0.90	0.83	9246	485	`PRICE`
1622	4	428	0	1.00	0.01	0.00	0.79	0.21	0.88	0.79	26	17	`ribotricer`
L. monocytogenes **and using overlap of 0.7**													
1337	263	169	285	0.82	0.61	0.18	0.89	0.11	0.85	0.78	0	0	`REPARATION_blast`
36	416	16	1586	0.02	0.96	0.98	0.69	0.31	0.04	0.22	21	12	`Ribo-TISH`
1554	159	273	68	0.96	0.37	0.04	0.85	0.15	0.90	0.83	2	0	`DeepRibo`
657	217	215	965	0.41	0.50	0.59	0.75	0.25	0.53	0.43	1	0	`IRSOM`
-	-	-	-	-	-	-	-	-	-	-	-	-	`SPECtre`
351	426	6	1271	0.22	0.99	0.78	0.98	0.02	0.35	0.38	0	0	`PRICE`
1622	4	428	0	1.00	0.01	0.00	0.79	0.21	0.88	0.79	1	0	`ribotricer`
L. monocytogenes **and using overlap of 0.9**													
1315	268	164	307	0.81	0.62	0.19	0.89	0.11	0.85	0.77	0	0	`REPARATION_blast`
21	421	11	1601	0.01	0.97	0.99	0.66	0.34	0.03	0.22	6	2	`Ribo-TISH`
1526	164	268	96	0.94	0.38	0.06	0.85	0.15	0.89	0.82	0	0	`DeepRibo`
657	217	215	965	0.41	0.50	0.59	0.75	0.25	0.53	0.43	0	0	`IRSOM`
-	-	-	-	-	-	-	-	-	-	-	-	-	`SPECtre`
342	429	3	1280	0.21	0.99	0.79	0.99	0.01	0.35	0.38	0	0	`PRICE`
1622	4	428	0	1.00	0.01	0.00	0.79	0.21	0.88	0.79	0	0	`ribotricer`

Table S7: Statistical evaluation of ORFs located in operons for the benchmark dataset *P. aeruginosa* and using overlap threshold of 0.01, 0.7 or 0.9. A detailed column description can be found in Table S1.

TP	TN	FP	FN	TPR	TNR	FNR	PPV	FDR	F1	accuracy	sTP	sFP	tool
P. aeruginosa **and using overlap of 0.01**													
1672	899	214	839	0.67	0.81	0.33	0.89	0.11	0.76	0.71	37	5	`REPARATION_blast`
1819	700	413	692	0.72	0.63	0.28	0.81	0.19	0.77	0.70	2196	234	`Ribo-TISH`
2473	716	397	38	0.98	0.64	0.02	0.86	0.14	0.92	0.88	8365	510	`DeepRibo`
1566	303	810	945	0.62	0.27	0.38	0.66	0.34	0.64	0.52	68	72	`IRSOM`
49	886	227	2462	0.02	0.80	0.98	0.18	0.82	0.04	0.26	0	0	`SPECtre`
2163	870	243	348	0.86	0.78	0.14	0.90	0.10	0.88	0.84	2274	26	`PRICE`
2404	180	933	107	0.96	0.16	0.04	0.72	0.28	0.82	0.71	19	9	`ribotricer`
P. aeruginosa **and using overlap of 0.7**													
1559	915	198	952	0.62	0.82	0.38	0.89	0.11	0.73	0.68	4	0	`REPARATION_blast`
81	1062	51	2430	0.03	0.95	0.97	0.61	0.39	0.06	0.32	43	30	`Ribo-TISH`
2353	948	165	158	0.94	0.85	0.06	0.93	0.07	0.94	0.91	361	25	`DeepRibo`
1524	332	781	987	0.61	0.30	0.39	0.66	0.34	0.63	0.51	3	7	`IRSOM`
49	886	227	2462	0.02	0.80	0.98	0.18	0.82	0.04	0.26	0	0	`SPECtre`
1356	991	122	1155	0.54	0.89	0.46	0.92	0.08	0.68	0.65	1	0	`PRICE`
2404	183	930	107	0.96	0.16	0.04	0.72	0.28	0.82	0.71	2	0	`ribotricer`
P. aeruginosa **and using overlap of 0.9**													
1446	926	187	1065	0.58	0.83	0.42	0.89	0.11	0.70	0.65	1	0	`REPARATION_blast`
41	1080	33	2470	0.02	0.97	0.98	0.55	0.45	0.03	0.31	9	5	`Ribo-TISH`
2298	965	148	213	0.92	0.87	0.08	0.94	0.06	0.93	0.90	81	7	`DeepRibo`
1522	333	780	989	0.61	0.30	0.39	0.66	0.34	0.63	0.51	0	4	`IRSOM`
49	886	227	2462	0.02	0.80	0.98	0.18	0.82	0.04	0.26	0	0	`SPECtre`
1002	1033	80	1509	0.40	0.93	0.60	0.93	0.07	0.56	0.56	0	0	`PRICE`
2404	183	930	107	0.96	0.16	0.04	0.72	0.28	0.82	0.71	0	0	`ribotricer`

Table S8: Statistical evaluation of ORFs located in operons for the benchmark dataset *S.* Typhimurium and using overlap threshold of 0.01, 0.7 or 0.9. A detailed column description can be found in Table S1.

TP	TN	FP	FN	TPR	TNR	FNR	PPV	FDR	F1	accuracy	sTP	sFP	tool
***S.* Typhimurium** and using overlap of **0.01**													
1848	579	429	99	0.95	0.57	0.05	0.81	0.19	0.87	0.82	58	25	`REPARATION_blast`
1235	841	167	712	0.63	0.83	0.37	0.88	0.12	0.74	0.70	1785	110	`Ribo-TISH`
1833	882	126	114	0.94	0.88	0.06	0.94	0.06	0.94	0.92	9535	56	`DeepRibo`
978	462	546	969	0.50	0.46	0.50	0.64	0.36	0.56	0.49	42	47	`IRSOM`
67	738	270	1880	0.03	0.73	0.97	0.20	0.80	0.06	0.27	2	2	`SPECtre`
1346	990	18	601	0.69	0.98	0.31	0.99	0.01	0.81	0.79	2298	2	`PRICE`
1846	341	667	101	0.95	0.34	0.05	0.73	0.27	0.83	0.74	44	36	`ribotricer`
***S.* Typhimurium** and using overlap of **0.7**													
1828	624	384	119	0.94	0.62	0.06	0.83	0.17	0.88	0.83	0	0	`REPARATION_blast`
174	949	59	1773	0.09	0.94	0.91	0.75	0.25	0.16	0.38	142	29	`Ribo-TISH`
1480	997	11	467	0.76	0.99	0.24	0.99	0.01	0.86	0.84	7	0	`DeepRibo`
964	470	538	983	0.50	0.47	0.50	0.64	0.36	0.56	0.49	2	6	`IRSOM`
67	739	269	1880	0.03	0.73	0.97	0.20	0.80	0.06	0.27	0	0	`SPECtre`
551	999	9	1396	0.28	0.99	0.72	0.98	0.02	0.44	0.52	0	0	`PRICE`
1844	347	661	103	0.95	0.34	0.05	0.74	0.26	0.83	0.74	0	0	`ribotricer`
***S.* Typhimurium** and using overlap of **0.9**													
1782	659	349	165	0.92	0.65	0.08	0.84	0.16	0.87	0.83	0	0	`REPARATION_blast`
121	968	40	1826	0.06	0.96	0.94	0.75	0.25	0.11	0.37	24	8	`Ribo-TISH`
1458	998	10	489	0.75	0.99	0.25	0.99	0.01	0.85	0.83	1	0	`DeepRibo`
963	470	538	984	0.49	0.47	0.51	0.64	0.36	0.56	0.48	1	1	`IRSOM`
67	739	269	1880	0.03	0.73	0.97	0.20	0.80	0.06	0.27	0	0	`SPECtre`
453	1000	8	1494	0.23	0.99	0.77	0.98	0.02	0.38	0.49	0	0	`PRICE`
1844	347	661	103	0.95	0.34	0.05	0.74	0.26	0.83	0.74	0	0	`ribotricer`

D.3 Stand-alone genes

Table S9: Statistical evaluation of ORFs located outside operons for the benchmark dataset *E. coli* and using overlap threshold of 0.01, 0.7, or 0.9. A detailed column description can be found in main Table S1.

TP	TN	FP	FN	TPR	TNR	FNR	PPV	FDR	F1	accuracy	sTP	sFP	tool
E. coli **and using overlap of 0.01**													
941	191	279	28	0.97	0.41	0.03	0.77	0.23	0.86	0.79	22	2	`REPARATION_blast`
624	376	94	345	0.64	0.80	0.36	0.87	0.13	0.74	0.69	907	60	`Ribo-TISH`
943	356	114	26	0.97	0.76	0.03	0.89	0.11	0.93	0.90	5892	178	`DeepRibo`
553	241	229	416	0.57	0.51	0.43	0.71	0.29	0.63	0.55	37	19	`IRSOM`
413	279	191	556	0.43	0.59	0.57	0.68	0.32	0.53	0.48	0	1	`SPECtre`
706	430	40	263	0.73	0.91	0.27	0.95	0.05	0.82	0.79	1711	13	`PRICE`
882	162	308	87	0.91	0.34	0.09	0.74	0.26	0.82	0.73	11	1	`ribotricer`
E. coli **and using overlap of 0.7**													
940	216	254	29	0.97	0.46	0.03	0.79	0.21	0.87	0.80	1	0	`REPARATION_blast`
30	447	23	939	0.03	0.95	0.97	0.57	0.43	0.06	0.33	21	14	`Ribo-TISH`
816	451	19	153	0.84	0.96	0.16	0.98	0.02	0.90	0.88	9	1	`DeepRibo`
516	256	214	453	0.53	0.54	0.47	0.71	0.29	0.61	0.54	5	3	`IRSOM`
413	279	191	556	0.43	0.59	0.57	0.68	0.32	0.53	0.48	0	0	`SPECtre`
105	453	17	864	0.11	0.96	0.89	0.86	0.14	0.19	0.39	1	0	`PRICE`
881	162	308	88	0.91	0.34	0.09	0.74	0.26	0.82	0.72	4	0	`ribotricer`
E. coli **and using overlap of 0.9**													
926	239	231	43	0.96	0.51	0.04	0.80	0.20	0.87	0.81	0	0	`REPARATION_blast`
14	454	16	955	0.01	0.97	0.99	0.47	0.53	0.03	0.33	2	2	`Ribo-TISH`
790	452	18	179	0.82	0.96	0.18	0.98	0.02	0.89	0.86	2	0	`DeepRibo`
516	256	214	453	0.53	0.54	0.47	0.71	0.29	0.61	0.54	1	1	`IRSOM`
413	279	191	556	0.43	0.59	0.57	0.68	0.32	0.53	0.48	0	0	`SPECtre`
77	454	16	892	0.08	0.97	0.92	0.83	0.17	0.15	0.37	0	0	`PRICE`
881	162	308	88	0.91	0.34	0.09	0.74	0.26	0.82	0.72	0	0	`ribotricer`

Table S10: Statistical evaluation of ORFs located outside operons for the benchmark dataset *L. monocytogenes* and using overlap threshold of 0.01, 0.7 or 0.9. A detailed column description can be found in Table S1.

TP	TN	FP	FN	TPR	TNR	FNR	PPV	FDR	F1	accuracy	sTP	sFP	tool
L. monocytogenes **and using overlap of 0.01**													
556	94	53	110	0.83	0.64	0.17	0.91	0.09	0.87	0.80	13	1	REPARATION_blast
300	99	48	366	0.45	0.67	0.55	0.86	0.14	0.59	0.49	223	28	Ribo-TISH
665	11	136	1	1.00	0.07	0.00	0.83	0.17	0.91	0.83	4595	514	DeepRibo
316	77	70	350	0.47	0.52	0.53	0.82	0.18	0.60	0.48	20	7	IRSOM
-	-	-	-	-	-	-	-	-	-	-	-	-	SPECtre
639	51	96	27	0.96	0.35	0.04	0.87	0.13	0.91	0.85	3418	188	PRICE
666	0	147	0	1.00	0.00	0.00	0.82	0.18	0.90	0.82	16	14	ribotricer
L. monocytogenes **and using overlap of 0.7**													
529	104	43	137	0.79	0.71	0.21	0.92	0.08	0.85	0.78	0	0	REPARATION_blast
19	140	7	647	0.03	0.95	0.97	0.73	0.27	0.05	0.20	11	6	Ribo-TISH
640	56	91	26	0.96	0.38	0.04	0.88	0.12	0.92	0.86	2	1	DeepRibo
305	81	66	361	0.46	0.55	0.54	0.82	0.18	0.59	0.47	5	1	IRSOM
-	-	-	-	-	-	-	-	-	-	-	-	-	SPECtre
110	139	8	556	0.17	0.95	0.83	0.93	0.07	0.28	0.31	0	0	PRICE
666	0	147	0	1.00	0.00	0.00	0.82	0.18	0.90	0.82	6	1	ribotricer
L. monocytogenes **and using overlap of 0.9**													
522	106	41	144	0.78	0.72	0.22	0.93	0.07	0.85	0.77	0	0	REPARATION_blast
15	143	4	651	0.02	0.97	0.98	0.79	0.21	0.04	0.19	1	0	Ribo-TISH
629	60	87	37	0.94	0.41	0.06	0.88	0.12	0.91	0.85	0	0	DeepRibo
305	81	66	361	0.46	0.55	0.54	0.82	0.18	0.59	0.47	3	1	IRSOM
-	-	-	-	-	-	-	-	-	-	-	-	-	SPECtre
106	141	6	560	0.16	0.96	0.84	0.95	0.05	0.27	0.30	0	0	PRICE
666	0	147	0	1.00	0.00	0.00	0.82	0.18	0.90	0.82	2	0	ribotricer

Table S11: Statistical evaluation of ORFs located outside operons for the benchmark dataset *P. aeruginosa* and using overlap threshold of 0.01, 0.7, or 0.9. A detailed column description can be found in main Table S1.

TP	TN	FP	FN	TPR	TNR	FNR	PPV	FDR	F1	accuracy	sTP	sFP	tool
P. aeruginosa **and using overlap of 0.01**													
781	427	98	643	0.55	0.81	0.45	0.89	0.11	0.68	0.62	26	1	`REPARATION_blast`
957	313	212	467	0.67	0.60	0.33	0.82	0.18	0.74	0.65	947	101	`Ribo-TISH`
1396	298	227	28	0.98	0.57	0.02	0.86	0.14	0.92	0.87	4650	280	`DeepRibo`
959	149	376	465	0.67	0.28	0.33	0.72	0.28	0.70	0.57	101	57	`IRSOM`
52	453	72	1372	0.04	0.86	0.96	0.42	0.58	0.07	0.26	0	0	`SPECtre`
1186	393	132	238	0.83	0.75	0.17	0.90	0.10	0.87	0.81	1006	19	`PRICE`
1335	93	432	89	0.94	0.18	0.06	0.76	0.24	0.84	0.73	4	1	`ribotricer`
P. aeruginosa **and using overlap of 0.7**													
712	435	90	712	0.50	0.83	0.50	0.89	0.11	0.64	0.59	2	0	`REPARATION_blast`
62	500	25	1362	0.04	0.95	0.96	0.71	0.29	0.08	0.29	36	6	`Ribo-TISH`
1336	430	95	88	0.94	0.82	0.06	0.93	0.07	0.94	0.91	300	20	`DeepRibo`
899	172	353	525	0.63	0.33	0.37	0.72	0.28	0.67	0.55	8	8	`IRSOM`
52	453	72	1372	0.04	0.86	0.96	0.42	0.58	0.07	0.26	0	0	`SPECtre`
765	456	69	659	0.54	0.87	0.46	0.92	0.08	0.68	0.63	2	0	`PRICE`
1334	93	432	90	0.94	0.18	0.06	0.76	0.24	0.84	0.73	1	0	`ribotricer`
P. aeruginosa **and using overlap of 0.9**													
651	439	86	773	0.46	0.84	0.54	0.88	0.12	0.60	0.56	0	0	`REPARATION_blast`
30	511	14	1394	0.02	0.97	0.98	0.68	0.32	0.04	0.28	3	0	`Ribo-TISH`
1273	441	84	151	0.89	0.84	0.11	0.94	0.06	0.92	0.88	46	8	`DeepRibo`
899	173	352	525	0.63	0.33	0.37	0.72	0.28	0.67	0.55	5	3	`IRSOM`
52	453	72	1372	0.04	0.86	0.96	0.42	0.58	0.07	0.26	0	0	`SPECtre`
541	485	40	883	0.38	0.92	0.62	0.93	0.07	0.54	0.53	0	0	`PRICE`
1334	93	432	90	0.94	0.18	0.06	0.76	0.24	0.84	0.73	1	0	`ribotricer`

Table S12: Statistical evaluation of ORFs located outside operons for the benchmark dataset *S.* Typhimurium and using overlap threshold of 0.01, 0.7, or 0.9. A detailed column description can be found in Table S1.

TP	TN	FP	FN	TPR	TNR	FNR	PPV	FDR	F1	accuracy	sTP	sFP	tool
***S.* Typhimurium** and using overlap of **0.01**													
1213	494	185	124	0.91	0.73	0.09	0.87	0.13	0.89	0.85	33	4	REPARATION_blast
797	609	70	540	0.60	0.90	0.40	0.92	0.08	0.72	0.70	1164	55	Ribo-TISH
1257	580	99	80	0.94	0.85	0.06	0.93	0.07	0.93	0.91	6083	54	DeepRibo
703	389	290	634	0.53	0.57	0.47	0.71	0.29	0.60	0.54	47	18	IRSOM
75	563	116	1262	0.06	0.83	0.94	0.39	0.61	0.10	0.32	1	1	SPECtre
866	654	25	471	0.65	0.96	0.35	0.97	0.03	0.78	0.75	1410	1	PRICE
1272	371	308	65	0.95	0.55	0.05	0.81	0.19	0.87	0.81	24	21	ribotricer
***S.* Typhimurium** and using overlap of **0.7**													
1189	544	135	148	0.89	0.80	0.11	0.90	0.10	0.89	0.86	0	0	REPARATION_blast
143	650	29	1194	0.11	0.96	0.89	0.83	0.17	0.19	0.39	116	13	Ribo-TISH
1041	660	19	296	0.78	0.97	0.22	0.98	0.02	0.87	0.84	12	1	DeepRibo
678	427	252	659	0.51	0.63	0.49	0.73	0.27	0.60	0.55	8	3	IRSOM
75	565	114	1262	0.06	0.83	0.94	0.40	0.60	0.10	0.32	0	0	SPECtre
349	677	2	988	0.26	1.00	0.74	0.99	0.01	0.41	0.51	0	0	PRICE
1270	385	294	67	0.95	0.57	0.05	0.81	0.19	0.88	0.82	0	0	ribotricer
***S.* Typhimurium** and using overlap of **0.9**													
1139	553	126	198	0.85	0.81	0.15	0.90	0.10	0.88	0.84	0	0	REPARATION_blast
97	658	21	1240	0.07	0.97	0.93	0.82	0.18	0.13	0.37	19	1	Ribo-TISH
1001	663	16	336	0.75	0.98	0.25	0.98	0.02	0.85	0.83	0	0	DeepRibo
678	427	252	659	0.51	0.63	0.49	0.73	0.27	0.60	0.55	2	0	IRSOM
75	565	114	1262	0.06	0.83	0.94	0.40	0.60	0.10	0.32	0	0	SPECtre
273	677	2	1064	0.20	1.00	0.80	0.99	0.01	0.34	0.47	0	0	PRICE
1270	385	294	67	0.95	0.57	0.05	0.81	0.19	0.88	0.82	0	0	ribotricer

D.4 Small Open Reading Frames

Table S13: Statistical evaluation of sORFs for the benchmark dataset *E. coli* and using overlap threshold of 0.01, 0.7 or 0.9. We could not find any sORF results for the benchmark dataset: *L. monocytogenes*. A detailed column description can be found in Table S1.

TP	TN	FP	FN	TPR	TNR	FNR	PPV	FDR	F1	accuracy	sTP	sFP	tool
E. coli **and using overlap of 0.01**													
23	50	10	31	0.43	0.83	0.57	0.70	0.30	0.53	0.64	0	0	REPARATION_blast
7	59	1	47	0.13	0.98	0.87	0.88	0.12	0.23	0.58	0	0	Ribo-TISH
50	50	10	4	0.93	0.83	0.07	0.83	0.17	0.88	0.88	55	8	DeepRibo
9	48	12	45	0.17	0.80	0.83	0.43	0.57	0.24	0.50	0	1	IRSOM
20	45	15	34	0.37	0.75	0.63	0.57	0.43	0.45	0.57	0	0	SPECtre
24	57	3	30	0.44	0.95	0.56	0.89	0.11	0.59	0.71	11	2	PRICE
50	51	9	4	0.93	0.85	0.07	0.85	0.15	0.88	0.89	11	1	ribotricer
E. coli **and using overlap of 0.7**													
18	51	9	36	0.33	0.85	0.67	0.67	0.33	0.44	0.61	0	0	REPARATION_blast
3	60	0	51	0.06	1.00	0.94	1.00	0.00	0.11	0.55	0	0	Ribo-TISH
44	53	7	10	0.81	0.88	0.19	0.86	0.14	0.84	0.85	3	0	DeepRibo
4	51	9	50	0.07	0.85	0.93	0.31	0.69	0.12	0.48	0	0	IRSOM
18	45	15	36	0.33	0.75	0.67	0.55	0.45	0.41	0.55	0	0	SPECtre
12	59	1	42	0.22	0.98	0.78	0.92	0.08	0.36	0.62	1	0	PRICE
47	52	8	7	0.87	0.87	0.13	0.85	0.15	0.86	0.87	3	0	ribotricer
E. coli **and using overlap of 0.9**													
18	52	8	36	0.33	0.87	0.67	0.69	0.31	0.45	0.61	0	0	REPARATION_blast
2	60	0	52	0.04	1.00	0.96	1.00	0.00	0.07	0.54	0	0	Ribo-TISH
42	53	7	12	0.78	0.88	0.22	0.86	0.14	0.82	0.83	2	0	DeepRibo
4	51	9	50	0.07	0.85	0.93	0.31	0.69	0.12	0.48	0	0	IRSOM
18	45	15	36	0.33	0.75	0.67	0.55	0.45	0.41	0.55	0	0	SPECtre
4	59	1	50	0.07	0.98	0.93	0.80	0.20	0.14	0.55	0	0	PRICE
47	52	8	7	0.87	0.87	0.13	0.85	0.15	0.86	0.87	0	0	ribotricer

Table S14: Statistical evaluation of sORFs for the benchmark dataset *P. aeruginosa* and using overlap threshold of 0.01, 0.7 or 0.9. We could not find any sORF results for the benchmark dataset: *L. monocytogenes*. A detailed column description can be found in Table S1.

TP	TN	FP	FN	TPR	TNR	FNR	PPV	FDR	F1	accuracy	sTP	sFP	tool
P. aeruginosa and using overlap of **0.01**													
5	4	1	2	0.71	0.8	0.29	0.83	0.17	0.77	0.75	0	0	REPARATION_blast
1	5	0	6	0.14	1.0	0.86	1.00	0.00	0.25	0.50	0	0	Ribo-TISH
7	4	1	0	1.00	0.8	0.00	0.88	0.12	0.93	0.92	6	2	DeepRibo
4	3	2	3	0.57	0.6	0.43	0.67	0.33	0.62	0.58	0	0	IRSOM
0	4	1	7	0.00	0.8	1.00	0.00	1.00	0.00	0.33	0	0	SPECtre
6	4	1	1	0.86	0.8	0.14	0.86	0.14	0.86	0.83	2	0	PRICE
5	2	3	2	0.71	0.4	0.29	0.62	0.38	0.67	0.58	0	0	ribotricer
P. aeruginosa and using overlap of **0.7**													
4	4	1	3	0.57	0.8	0.43	0.80	0.20	0.67	0.67	0	0	REPARATION_blast
0	5	0	7	0.00	1.0	1.00	0.00	0.00	0.00	0.42	0	0	Ribo-TISH
7	4	1	0	1.00	0.8	0.00	0.88	0.12	0.93	0.92	0	1	DeepRibo
3	4	1	4	0.43	0.8	0.57	0.75	0.25	0.55	0.58	0	0	IRSOM
0	4	1	7	0.00	0.8	1.00	0.00	1.00	0.00	0.33	0	0	SPECtre
4	5	0	3	0.57	1.0	0.43	1.00	0.00	0.73	0.75	0	0	PRICE
4	2	3	3	0.57	0.4	0.43	0.57	0.43	0.57	0.50	0	0	ribotricer
P. aeruginosa and using overlap of **0.9**													
4	4	1	3	0.57	0.8	0.43	0.80	0.20	0.67	0.67	0	0	REPARATION_blast
0	5	0	7	0.00	1.0	1.00	0.00	0.00	0.00	0.42	0	0	Ribo-TISH
6	4	1	1	0.86	0.8	0.14	0.86	0.14	0.86	0.83	0	0	DeepRibo
3	4	1	4	0.43	0.8	0.57	0.75	0.25	0.55	0.58	0	0	IRSOM
0	4	1	7	0.00	0.8	1.00	0.00	1.00	0.00	0.33	0	0	SPECtre
4	5	0	3	0.57	1.0	0.43	1.00	0.00	0.73	0.75	0	0	PRICE
4	2	3	3	0.57	0.4	0.43	0.57	0.43	0.57	0.50	0	0	ribotricer

Table S15: Statistical evaluation of sORFs for the benchmark dataset *S.* Typhimurium and using overlap threshold of 0.01, 0.7 or 0.9. We could not find any sORF results for the benchmark dataset: *L. monocytogenes*. A detailed column description can be found in Table S1.

TP	TN	FP	FN	TPR	TNR	FNR	PPV	FDR	F1	accuracy	sTP	sFP	tool
***S.* Typhimurium and using overlap of 0.01**													
11	62	7	20	0.35	0.90	0.65	0.61	0.39	0.45	0.73	0	0	`REPARATION_blast`
4	64	5	27	0.13	0.93	0.87	0.44	0.56	0.20	0.68	0	0	`Ribo-TISH`
30	59	10	1	0.97	0.86	0.03	0.75	0.25	0.85	0.89	38	11	`DeepRibo`
6	48	21	25	0.19	0.70	0.81	0.22	0.78	0.21	0.54	1	1	`IRSOM`
3	62	7	28	0.10	0.90	0.90	0.30	0.70	0.15	0.65	0	0	`SPECtre`
18	65	4	13	0.58	0.94	0.42	0.82	0.18	0.68	0.83	8	0	`PRICE`
30	54	15	1	0.97	0.78	0.03	0.67	0.33	0.79	0.84	1	4	`ribotricer`
***S.* Typhimurium and using overlap of 0.7**													
10	68	1	21	0.32	0.99	0.68	0.91	0.09	0.48	0.78	0	0	`REPARATION_blast`
2	68	1	29	0.06	0.99	0.94	0.67	0.33	0.12	0.70	0	0	`Ribo-TISH`
26	64	5	5	0.84	0.93	0.16	0.84	0.16	0.84	0.90	1	0	`DeepRibo`
5	58	11	26	0.16	0.84	0.84	0.31	0.69	0.21	0.63	0	0	`IRSOM`
3	64	5	28	0.10	0.93	0.90	0.38	0.62	0.15	0.67	0	0	`SPECtre`
9	68	1	22	0.29	0.99	0.71	0.90	0.10	0.44	0.77	0	0	`PRICE`
29	58	11	2	0.94	0.84	0.06	0.72	0.28	0.82	0.87	0	0	`ribotricer`
***S.* Typhimurium and using overlap of 0.9**													
10	68	1	21	0.32	0.99	0.68	0.91	0.09	0.48	0.78	0	0	`REPARATION_blast`
1	68	1	30	0.03	0.99	0.97	0.50	0.50	0.06	0.69	0	0	`Ribo-TISH`
24	65	4	7	0.77	0.94	0.23	0.86	0.14	0.81	0.89	0	0	`DeepRibo`
5	58	11	26	0.16	0.84	0.84	0.31	0.69	0.21	0.63	0	0	`IRSOM`
3	64	5	28	0.10	0.93	0.90	0.38	0.62	0.15	0.67	0	0	`SPECtre`
2	68	1	29	0.06	0.99	0.94	0.67	0.33	0.12	0.70	0	0	`PRICE`
29	58	11	2	0.94	0.84	0.06	0.72	0.28	0.82	0.87	0	0	`ribotricer`

D.5 Novel sORF detection

Table S16: Numbers of novel sORFs detected for all tools. The study of Weaver *et al* [4] experimentally identified 31 novel sORFs. The novel sORF detection power for each tool was evaluated by applying our benchmark pipeline on the *E. coli* Ribo-seq library described in [4]. Note: `DeepRibo` predicted in total 17 sORFs, but using a cutoff 18 sORFs remain. Since no RNA library is available we could not test novel sORF detection for `IRSOM`.

Tool	`DeepRibo`	`REPARATION_blast`	`Ribo-TISH`	`SPECtre`	`smORFer`	`PRICE`	`ribotricer`
Novel sORFs	17	0	0	0	28	0	0

E Evaluation of key results

To follow the good practice for benchmarking as proposed by Mangul *et al.* [5] we adapted one of their example summary figures [6] for our benchmark scenario. Here, we propose a straightforward evaluation system to summarize the performance of all evaluated tools. The evaluation results are visualized in Figure 5 of the main text. The evaluation system reveals the performance of each tool for several categories. We rate the tools as follows: a violet circle for superior performance, a light blue circle for satisfactory performance, and a dark blue circle for unsatisfactory performance. How each tool is rated for each category is described in the following.

E.1 Predictive power for the *translatome* set

To evaluate the predictive power of each tool, we averaged the AUC values of the PRC of all *translatome* benchmark sets. `Ribo-TISH`, `IRSOM`, `PRICE` and `ribotricer` achieved unsatisfactory results, with average AUCs of 0.69, 0.70, 0.76 and 0.72 , respectively.`REPARATION_blast`, with an average AUC of 0.88, were satisfactory. Since `smORFer` only has an AUC for *E.coli* of 0.82 was also satisfactory and `DeepRibo` achieved a superior predictive power, with AUCs of 0.94. The AUC values can be found in Table 3 of the main document.

Superior	:	AUC higher on average 0.90
Satisfactory	:	AUC higher on average 0.80
Unsatisfactory	:	AUC lower on average 0.80

E.2 Predictive power inside and outside of operons

Averaging the AUCs for the detection of ORFs inside or outside of operons, respectively, led to the following results: `Ribo-TISH` 0.67 and 0.72, `DeepRibo`

0.93 and 0.95, REPARATION_blast 0.91 and 0.91, IRSOM 0.68 and 0.75, SPECtre 0.53 and 0.7, smORFer 0.81 and 0.84, PRICE 0.76 and 0.79, ribotricer 0.66 and 0.63.

Superior	: AUC higher on average 0.90
Satisfactory	: AUC higher on average 0.80
Unsatisfactory	: AUC lower on average 0.80

E.3 Prediction of novel sORFs

The predictive power of finding novel ORFs was tested using 33 verified novel ORFs outside of the annotation([7], see main text). Only DeepRibo was able to find 18/19 of the 33 novel ORFs and has therefore a Satisfactory predictive power. REPARATION_blast with only two and Ribo-TISH and IRSOM with which found non of the novel ORFs show a unsatisfactory performance.

Superior	: 20 novel ORFs detected
Satisfactory	: 10 novel ORFs detected
Unsatisfactory	: less than 10 novel ORFs detected

E.4 Runtime

The runtime comparison can be found in the main document (Table 7). We evaluated tools using single- and multithreading. Since not every tool supports multithreading, we took the minimum runtime for each tool for either single- or multithreading. Be aware that the original REPARATION tool should be faster than REPARATION_blast because of the use of ublast in REPARATION_blast.

Superior	if test data was computed in less than 30 minutes
Satisfactory	if test data was computed in less than 2 hours
Unsatisfactory	if test data was computed in more than 2 hours

E.5 Memory

The memory comparison can be found in the main document (Table 7). We evaluated tools using single- and multithreading. Since not every tool supports multithreading, we take the minimum run time for each tool from either approach.

Superior	: 2 or less GB
Satisfactory	: 4 or less GB
Unsatisfactory	: more than 4 GB

E.6 Applicability

The applicability describes how universally the tool can be applied. In the following we list several applicability criteria. If a tools fulfills the criteria it gets one point. Based on the amount of points each tool achieved the applicability is evaluated:

1. Can use replicates
2. Is deterministic
3. Outputs a standard file format (gff/bed/ ...)
4. Uses unit testing or some other correctness declaration
5. Stable results throughout different organisms

In the following, we describe how each tool was rated for applicability:

Table S17: Applicability scoring table showing: (1) Can use replicates, (2) Is deterministic, (3) Outputs a standard file format, (4) Uses unit testing or some other correctness declaration, (5) Stable results throughout different organisms.

tool	1	2	3	4	5	total
Ribo-TISH	1	1	0	0	1	3
DeepRibo	0	1	1	0	1	3
REPARATION_blast	0	0	0	0	1	1
IRSOM	0	1	0	0	1	2
SPECtre	0	1	0	0	0	2
smORFer	0	1	1	0	-	2
PRICE	0	0	1	1	0	2
ribotricer	0	1	0	0	1	2

Superior : 4 or more points fulfilled
Satisfactory : 2 or more points fulfilled
Unsatisfactory : 1 or less points fulfilled

E.7 Usability

User friendliness or usability is one of the key factors on how convenient it is for the users to apply the tool. In the following, we describe several usability criteria, each giving a single point if it is fulfilled by the tested tool.

1. Hosting software on a website with predicted long-term accessibility (e.g., GitHub)
2. Installation via package managers (e.g., Bioconda)
3. Provides an example dataset for testing
4. Version control (changelog)
5. Documentation: parameters

6. Documentation: input
7. Documentation: output
8. Documentation: dependencies
9. Open source

In the following, we describe the rating of the tools' usability:

Table S18: Usability scoring table showing: (1) Long-term accessible website, (2) Package managers, (3) Example data, (4) Version control, (5) Documentation: parameter, (6) Documentation: input, (7) Documentation: output, (8) Documentation: dependencies, (9) Open source. * *REPARATION* was using a proprietary, closed source sequence search tool that we replaced with the free and open source tool blast, to make the software usable without fee. This version is called `REPARATION_blast`.

tool	1	2	3	4	5	6	7	8	9	total
`Ribo-TISH`	1	1	0	1	1	1	1	1	1	8
`DeepRibo`	1	0	1	1	1	0	1	1	1	7
`REPARATION_blast`	1	1	1	0	1	1	0	1	1*	7
`IRSOM`	1	0	1	0	0	1	0	1	1	5
`SPECtre`	1	0	1	1	1	1	1	1	1	8
`smORFer`	1	0	1	0	0	1	1	0	1	5
`PRICE`	1	0	0	1	1	1	1	1	1	7
`ribotricer`	1	1	0	1	1	1	1	1	1	8

Superior	:	8 or more points fulfilled
Satisfactory	:	4 or more points fulfilled
Unsatisfactory	:	3 or less points fulfilled

F Adapter sequences used for trimming

F.1 *E. coli*

AGATCGGAAGAGCACACGTCTGAACTCCAGTCAC

F.2 *L. monocytogenes*

AGATCGGAAGAGCACACGTCTGAACTCCAGTCAC

F.3 *P. aeruginosa*

AGATCGGAAGAGCACACGTCTGAACTCCAGTCAC

F.4 *S.* Typhimurium

CTGTAGGCACCATCAATAGATCGGAAGA

References

1. Jeremy Weaver, Fuad Mohammad, Allen R Buskirk, and Gisela Storz. Identifying small proteins by ribosome profiling with stalled initiation complexes. *mBio*, 10(2):e02819–18, 2019.
2. Rondine J Allen, Evan P Brenner, Caitlin E VanOrsdel, Jessica J Hobson, David J Hearn, and Matthew R Hemm. Conservation analysis of the CydX protein yields insights into small protein identification and evolution. *BMC genomics*, 15(1):946, 2014.
3. Chelsea R Lloyd, Seongjin Park, Jingyi Fei, and Carin K Vanderpool. The small protein SgrT controls transport activity of the glucose-specific phosphotransferase system. *Journal of bacteriology*, 199(11):e00869–16, 2017.
4. Jeremy Weaver, Fuad Mohammad, Allen R Buskirk, and Gisela Storz. Identifying small proteins by ribosome profiling with stalled initiation complexes. *MBio*, 10(2):e02819–18, 2019.
5. Serghei Mangul, Lana S Martin, Eleazar Eskin, and Ran Blekhman. Improving the usability and archival stability of bioinformatics software, 2019.
6. Keegan Korthauer, Patrick K Kimes, Claire Duvallet, Alejandro Reyes, Ayshwarya Subramanian, Mingxiang Teng, Chinmay Shukla, Eric J Alm, and Stephanie C Hicks. A practical guide to methods controlling false discoveries in computational biology. *Genome biology*, 20(1):118, 2019.
7. Lukas M Weber, Wouter Saelens, Robrecht Cannoodt, Charlotte Soneson, Alexander Hapfelmeier, Paul P Gardner, Anne-Laure Boulesteix, Yvan Saeys, and Mark D Robinson. Essential guidelines for computational method benchmarking. *Genome biology*, 20(1):125, 2019.
